# Hypertension and Atrial Fibrillation: A Study on Epidemiology and Mendelian Randomization Causality

**DOI:** 10.3389/fcvm.2021.644405

**Published:** 2021-03-23

**Authors:** Li-Zhen Liao, Xiu-Yun Wen, Shao-Zhao Zhang, Wei-Dong Li, Xiao-Dong Zhuang

**Affiliations:** ^1^Guangzhou Higher Education Mega Center, Guangdong Pharmaceutical University, Guangzhou, China; ^2^Guangdong Engineering Research Center for Light and Health, Guangzhou Higher Education Mega Center, Guangzhou, China; ^3^Guangdong Key Laboratory of Pharmaceutical Bioactive Substances, Guangdong Pharmaceutical University, Guangzhou, China; ^4^The First Affiliated Hospital of Sun Yat-sen University, Guangzhou, China

**Keywords:** hypertension, atrial fibrillation, mendelian randomization, genome-wide association study, causality

## Abstract

**Introduction:** Hypertension (HT) and atrial fibrillation (AF) often coexist. However, the causality between these two conditions remains to be determined.

**Methods:** We used individual participant data from the Atherosclerosis Risk in Communities (ARIC) prospective cohort with 9,474 participants. HT was ascertained at visit 1 (1987–1989), and incident AF was identified by ECGs conducted during study examinations at each visit, hospital discharge codes, and death certificates. We used the Kaplan–Meier estimate to compute the cumulative incidence of AF by the HT subgroup. Then we used Cox hazard regression model to assess the association between HT and incident AF. The causality between genetically determined HT and AF was analyzed by the two-sample Mendelian randomization (MR) based on publicly summarized genome-wide association studies (GWASs) data.

**Results:** A total of 1,414 cases (14.9%) of AF were identified during the follow-up period (median 24.1 years). After adjusting for all covariates, the hazard ratio between the participants with HT and incident AF was 1.50 [95% confidence interval (CI) 1.29–1.73]. In the HT → AF MR analysis, we detected a causal correlation between HT and AF (OR: 1.90, 95% CI 1.18–3.04, *P* = 0.01) with no evidence of heterogeneity from single-nucleotide polymorphisms. Besides, the genetically determined SBP and DBP (10 mmHg) were consistently associated with a higher risk of AF.

**Conclusions:** In the ARIC study, the incident AF increased by 50% in patients with HT. In the MR analysis, our results supported causal inference between HT and AF.

## A Key Messages Box

In epidemiological studies, the incidence of atrial fibrillation (AF) increased by 50% in patients with hypertension (HT). HT might be a genetic cause of AF.

## Introduction

Hypertension (HT) and atrial fibrillation (AF) are two important public health priorities. Their prevalence is increasing worldwide, and the two conditions often coexist in the same patients (1). Both conditions are associated with aging. Additionally, HT is also related to other cardiovascular comorbidities that increase the risk of AF, including coronary heart disease (CHD), heart failure (HF), metabolic syndrome, chronic kidney disease, and sleep apnea (2). The epidemiological association between HT and AF was established in many previous studies (3–5). In patients with established AF, their HT morbidity was reportedly much higher than that of non-AF (3). Moreover, following HF, aging, and valvular heart disease, HT portends an excess risk of AF by 50% in men and 40% in women (4). Considering the high prevalence of HT in the population, it accounts for more cases of AF than other risk factors (6, 7).

Despite the well-established epidemiological association between HT and AF, these preliminary observational data were limited for causal inference due to the potential bias introduced by confounding factors and reverse causality (3, 7). Hence, understanding the causal relation between HT and AF has important public health significance for disease prevention and complication management. Mendelian randomization (MR) is a robust genetic methodology used to identify causal risk factors for diseases (8). It relies on three main assumptions, which are shown in Supplementary Figure 1 (9). Subject to a genetic variant satisfying the instrumental variable assumptions, an association between the variant and outcome implied a causal effect of the exposure on the outcome. In this study, our goal was to describe the association between HT and AF in a considerable prospective cohort Atherosclerosis Risk in Communities (ARIC) study. We also conducted a two-sample MR analysis for the causal relationship between HT and AF and systolic blood pressure (SBP), diastolic blood pressure (DBP), and AF.

## Methods

### Ethics

The studies involving human participants were reviewed and approved by the ethics committee of Guangdong Pharmaceutical University. The participants provided written informed consent to participate in this study.

### Study Population

The ARIC study design was previously described (10). A total of 15,792 participants, aged 45 to 64 years, were recruited between 1987 and 1989 (visit 1). Later on, there were four subsequent study visits in 1990–1992 (visit 2), 1993–1995 (visit 3), 1996–1998 (visit 4), and 2011–2013 (visit 5). We excluded participants with prevalent AF or missing follow-up data, HT data, and other covariates. A total of 9,474 participants were eventually included in our analysis.

### HT and AF Assessment

HT was ascertained at visit 1 (a measured SBP ≥140 mmHg and/or DBP ≥ 90 mmHg). Three blood pressure measurements were obtained from seated participants with a 5-min rest period. The average of the second and third measurements was recorded.

Incident AF was identified by the following three methods, (1) electrocardiograms (ECGs), (2) hospital discharge codes, and (3) death certificates. Twelve-lead ECGs were conducted with participants lying in a supine position at each visit. ECGs were automatically coded as a cardiologist confirmed AF. ECG data were transmitted electronically to a reading center (EpiCare, Wake Forest University, Winston-Salem, NC, USA), reviewed, and analyzed using the GE Marquette 12-SL program (GE Marquette, Milwaukee, WI, USA). Trained abstractors collected information from all participant hospitalizations using the International Classification of Diseases, Ninth Revision, Clinical Modification (ICD-9-CM) codes for diagnoses. AF was ascertained if the ICD-9-CM code 427.31 (AF) or 427.32 (atrial flutter) was present in any hospitalization. AF associated with open cardiac surgery was excluded. AF was also defined if ICD-9-CM codes 427.31 or 427.32 were listed as a cause of death (11).

### Measurement of Other Covariates

All of the covariates such as race, gender, and age were assessed at visit 1. The educational level was self-reported. Physical activity was accessed using the validated Baecke questionnaire. Height and weight were measured with the participants wearing light clothes. Body mass index (BMI) was calculated as weight (in kilograms) divided by squared height (in meters). Diabetes was defined as fasting blood glucose ≥126 mg/dl, non-fasting blood glucose ≥200 mg/dl, use of antidiabetic medicine, or self-reported physician diagnosis of diabetes. Total cholesterol, high-density lipoprotein cholesterol (HDL-c), and triglycerides (TG) were measured using standardized enzymatic assays, and low-density lipoprotein (LDL-c) was calculated based on the Friedewald formula. Creatine was measured using a modified kinetic Jaffe method (10). Stroke, CHD, and HF were defined as previously described (12–14).

### Summary of Genome-Wide Association Studies (GWAS) Data

Data included in this MR study were the GWAS summary statistics datasets from the Neale Lab consortium for HT, SBP, and DBP, and the Ben Elsworth consortium for AF. Details of the studies and datasets used for the analyses are presented in Table 1.

**Table 1:** Details of studies and datasets used for the MR analyses.

**Exposure/outcome**	**Participants**	**Sample size**	**Data source**	**First author**	**Consortium**	**Year**	**Units**
HT	European, males, and females	337,199	http://www.nealelab.is/blog/2017/9/11/details-and-considerations-of-the-uk-biobank-gwas	Neale	Neale Lab	2017	NA
SBP	European, males, and females	317,754	http://www.nealelab.is/blog/2017/9/11/details-and-considerations-of-the-uk-biobank-gwas	Neale	Neale Lab	2017	10 mmHg
DBP	European, males, and females	317,756	http://www.nealelab.is/blog/2017/9/11/details-and-considerations-of-the-uk-biobank-gwas	Neale	Neale Lab	2017	10 mmHg
AF	European, males, and females	463,010	41202#I48: Output from GWAS pipeline using Phesant derived variables from UKBiobank	Ben Elsworth	MRC-IEU	2018	NA

*MR, Mendelian randomization; HT, Hypertension; SBP, Systolic blood pressure; DBP, Diastolic blood pressure; AF, Atrial fibrillation; NA, not available*.

### Data Extraction and Harmonization

We requested the following SNP genotype quality metrics from disease and risk factor studies: strong evidence of between-study heterogeneity in the SNP-trait association (*P* ≤ 0.001), Hardy–Weinberg disequilibrium (*P* ≤ 0.001), or imputation quality metric (info or *r*^2^) ≤ 0.90. We harmonized the summary data for diseases and risk factors so that the allele effect reflected the alleles associated with exposure. When SNPs were palindromic, A/T or G/C, we used information on the allele frequency to resolve strand ambiguity. We excluded SNP–trait associations from the GWAS catalog if they missed a *P*-value, beta, or an SE for the beta. The included SNPs are shown in Supplementary Tables 1–3.

### Mendelian Randomization Analysis for Genetic Causality Assessment

Since MR for the SNP exposure effects and SNP outcome effects were obtained from separate studies, it was possible to estimate the causal influence of the exposure on the outcome (9). Our MR study was conducted on the MR-Base platform online (http://www.mrbase.org). We conducted two-sample MR approaches for the genetic causality assessment (HT → AF, SBP → AF, and DBP → AF) using publicly available summarized data from the GWAS (15).

### Statistical Analysis

For the ARIC study, baseline characteristics between the HT subgroups were compared using one-way ANOVA, the χ^2^-test, and the Kruskal–Wallis test as appropriate. The Kaplan–Meier estimate was used to compute the cumulative incidence of AF by the HT subgroups, and differences in estimates were compared using the log-rank procedure. Cox hazard regression models were used to assess the association between HT and incident AF. The follow-up time was defined as the time from baseline (visit 1) to outcome occurrence, loss to follow-up, death, or December 31, 2014, whichever occurred first. Pre-specified subgroup analyses were performed by gender, age, race, smoking, drinking, BMI, creatine, LDL-c, TG, and potential interactions with HT. We also conducted sensitivity analyses, excluding participants with HF, CHD, and diabetes. Cox hazard regression models were also used to assess the association between SBP or DBP and AF separately as continuous variables. We also used a restricted cubic spline with three knots to explore the potential non-linear trends for SBP and DBP hazard ratios, respectively.

For the MR analysis, the strength of the instrumental variables was assessed using the F statistic. Causality between genetically determined HT, SBP, DB, and AF was estimated. Using the HT → AF MR analysis as an example, each SNP's association with AF was weighted by its association with HT, and estimates were combined using an inverse variance weighted (IVW) method (16). Several sensitivity analyses were carried out, including (1) the weighted median method, (2) the weighted mode method, (3) MR-Egger regression, (4) funnel plots, and (5) leave-one-out analysis.

All of the statistical tests were two-sided. The statistical test for the MR analyses was considered statistically significant at *P* < 0.05. All of the analyses were conducted using Stata (version 14.2, StataCorp LP, College Station, TX, USA) and R (version 3.2.5, R Foundation for Statistical Computing, Vienna, Austria) (16).

## Results

### Hypertension, Systolic Blood Pressure, Diastolic Blood Pressure, and Incident Atrial Fibrillation

The baseline characteristics are shown in Supplementary Table 4. Of the 9,474 participants, 1,190 had HT. Cases, 1,414 (14.9%), of AF occurred during a median 24.1 follow-up years. Unadjusted cumulative curves for incident AF are demonstrated in Supplementary Figure 2. Restricted cubic spline showed an increasing linear risk for SBP and a potential U-shaped risk tendency for DBP (Supplementary Figures 3, 4). In the adjusted model, the participants with HT were associated with a 50% increased rate of incident AF [hazard ratio, 1.50; 95% confidence interval (CI), 1.29–1.73] (Supplementary Table 5). The hazard ratios of SBP and DBP for incident AF were 1.17 (95% CI, 1.12–1.22) and 0.90 (95% CI, 0.84, 0.97), respectively, after adjusting for each other in the final models (Supplementary Table 5). The results were similar when stratified by sex, race, smoking, drinking, BMI, creatine, LDL-c, and TG in the subgroup analyses (all P_interaction_ > 0.05, Supplementary Figure 5). After excluding the participants with HF, CHD, or diabetes, the association between HT and incident AF persisted (Supplementary Figure 5). To summarize, the HT participants were associated with an increased AF incident rate.

### Causal Associations Between Genetically Determined Hyperstension, Systolic Blood Pressure, Diastolic Blood Pressure, and Atrial Fibrillation

**Figure 1:**
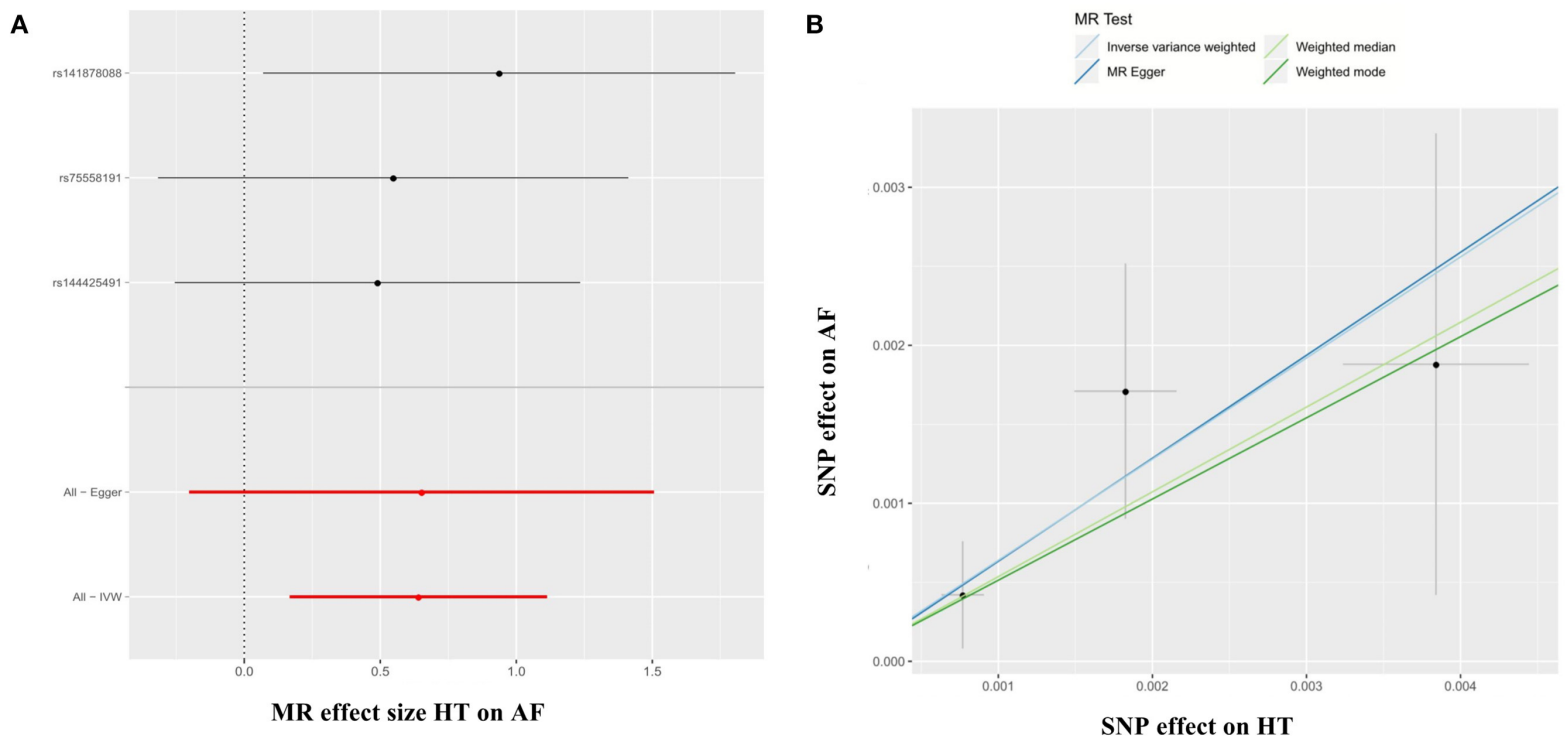
Forest plots **(A)** and scatter plots **(B)** of causal effects between HT-associated SNPs and risk of AF. The slopes of each line in the scatter plots represent the causal association for each method. MR, Mendelian randomization; SNP, single-nucleotide polymorphism; HT, hypertension; AF, atrial fibrillation; IVW, inverse variance weighted.

The 3 HT-associated SNPs explained 1.04% of the variance in the AF levels, and the mean F statistic was 68. In the HT → AF MR analysis using the conventional method (inverse variance weighted, IVW), we detected a causal relationship between HT and AF [odds ratio (OR): 1.90, 95% CI: 1.18–3.04, P = 0.01) with no evidence of heterogeneity between estimates from individual SNPs [P_heterogeneity_ = 0.42 (MR-Egger) and P_heterogeneity_ = 0.72 (IVW)] and the pleiotropy effect (P_pleiotropy_ = 0.97) (Supplementary Table 6, Figures 1A,B).

In the SBP → AF and DBP → AF MR analyses using the IVW method, our MR analyses showed that the genetically determined SBP and DBP were consistently associated with a higher risk of AF (SBP → AF, OR: 1.03, 95% CI: 1.01–1.05, P = 0.01; DBP → AF, OR: 1.02, 95% CI: 1.00–1.04, P = 0.03) with no evidence of heterogeneity between estimates from individual SNPs or the pleiotropy effect [SBP → AF, P_heterogeneity_ = 0.88 (MR-Egger), P_heterogeneity_ = 0.88 (IVW), and P_pleiotropy_ = 0.46; DBP → AF, P_heterogeneity_ = 0.72 (MR-Egger), P_heterogeneity_ = 0.73 (IVW), and P_pleiotropy_ = 0.50) (Supplementary Table 6, Supplementary Figures 6A,B, 7A,B). The results were the same as in the weighted median and weighted mode methods in the SBP → AF analysis (all *P* < 0.05). In the DBP → AF analysis, the results were similar in the weighted mode method (*P* = 0.02) (Supplementary Table 6).

**Figure 2:**
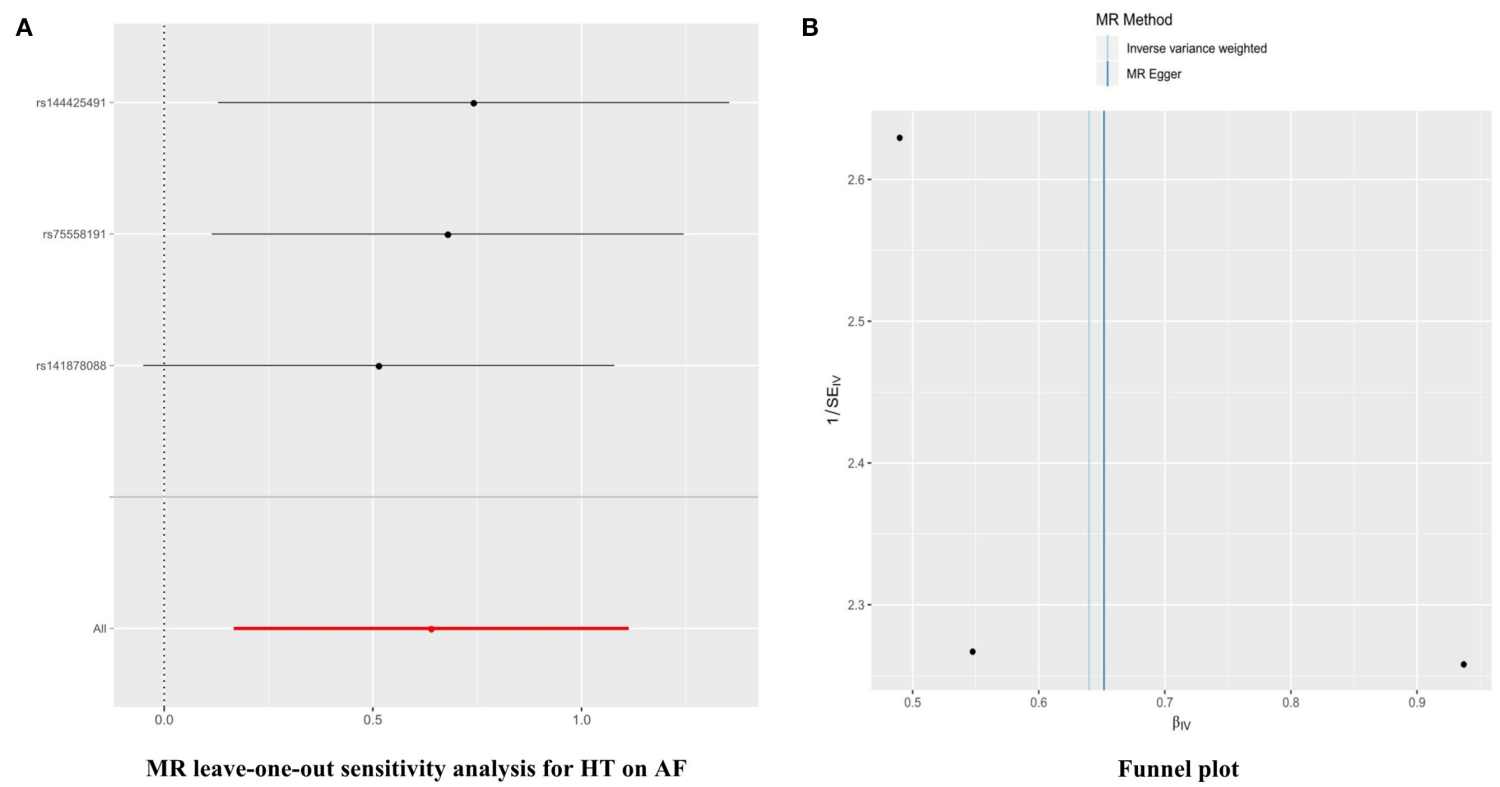
Leave-one-out sensitivity analysis and funnel plots in the HT → AF MR analysis. **(A)** Leave-one-out sensitivity analysis. Each black point represents the IVW MR method applied to estimate the causal effect of HT on AF, excluding that particular variant from the analysis. The red point depicts the IVW estimate using all of the SNPs. There are no instances where the exclusion of one particular SNP leads to dramatic changes in the overall result. **(B)** Funnel plot of the relationship between the causal effect of HT on AF. Funnel plot showing the relationship between the causal effect of HT on AF estimated using each SNP as a separate instrument against the inverse of the standard error of the causal estimate. Vertical lines show the causal estimates using all of the SNPs combined into a single instrument for the two different methods. Asymmetry in the funnel plot may be indicative of violations of the assumption through horizontal pleiotropy. MR, Mendelian randomization; SNP, single-nucleotide polymorphism; HT, hypertension; AF, atrial fibrillation; IVW, inverse variance weighted.

Furthermore, in the leave-one-out analysis, we found that no single instrument was strongly driving the overall effect of HT → AF (Figure 2A). There was no funnel plot asymmetry (Figure 2B). Both the leave-one-out analysis and funnel plots further suggested that no SNPs exhibited horizontal pleiotropy. The horizontal pleiotropy results were similar in the SBP/DBP → AF analysis (Supplementary Figures 8A,B, 9A,B).

## Discussion

In a large-scale ARIC cohort, we demonstrated that the participants with HT were associated with a 50% increase in incident AF. In the MR analysis, our results supported causal inference between HT and AF. These findings highlight the importance of optimal blood pressure control in the HT population to prevent AF. Therapeutics targeting HT treatment are likely to prevent AF effectively.

Previous epidemiological studies revealed that HT was an established risk factor for new-onset AF (4, 17, 18). However, these findings were limited in demonstrating a causal role for HT in AF development due to the potential of residual confounding and reverse causation (19). Previously, also in the ARIC study, it was reported that overall, 56.5% of AF cases could be explained by having ≥1 elevated risk factors, of which elevated blood pressure was the most important contributor (7). In our study, after adjusting for all of the covariates, the results indicated that the HT participants were associated with a 50% increased rate of incident AF. It was consistent with the previous study demonstrating the relationship between HT and AF (7). However, in our study, we had a much longer follow-up time (a median 24.1 years), while the mean follow-up time is 17.1 years in the previous survey. Moreover, our aim was different, resulting in different patient classification. In our study, our goal was to describe the association between HT and AF in the ARIC study, so patients were divided into two groups according to whether they had HT or not in visit 1. In the previous ARIC study, individuals were classified into three groups (optimal, borderline, and elevated level), according to the established AF risk factors (high blood pressure, elevated body mass index, diabetes, cigarette smoking, and prior cardiac disease) (7). Furthermore, in our study, the hazard ratios of SBP and DBP for incident AF were 1.17 (95% CI, 1.12–1.22) and 0.90 (95% CI, 0.84, 0.97), respectively, after adjusting for each other in the final models (Supplementary Table 5). We speculated that the effect of HT on AF was primarily through SBP instead of DBP.

Nowadays, the most common sustained cardiac arrhythmia is AF. It is associated with substantial healthcare use, stroke, and mortality. Significant strides have been made in stroke prevention and rhythm control strategies, yet reducing the incidence of AF has been slowed by increasing the incidence and prevalence of AF risk factors, including obesity, physical inactivity, sleep apnea, diabetes mellitus, HT, and other modifiable lifestyle-related factors (20). Since HT is the most important modifiable risk factor for AF, and abundant previous evidence supported the association between HT and AF, we were eager to know whether HT served as an etiology for AF. So we conducted the MR study to test the causality between HT and AF. Our results provided evidence supporting a causal association between genetically determined HT and AF [odds ratio (OR): 1.90, 95% CI: 1.18–3.04, *P* = 0.01). Two prior studies are looking at blood pressure genetics and AF (5, 21). The first one demonstrated that SBP and DBP mediated ischemic stroke risk, in part, through AF (21). The second one found that blood pressure was associated with increased risk of AF, and blood pressure reduction with calcium channel blockade or beta-blockade could reduce the risk of AF in another consortium, which was different from ours (5).

Several pathophysiologic mechanisms could explain the relationship between HT and AF (22–24). HT animal models elaborated that high blood pressure led to left atrial scaring and inflammation (22, 23), and then, it would create altered patterns of conduction and functional slowing, resulting in AF development and perpetuation (23, 24). Moreover, HT-induced neurohormonal activation and autonomic dysfunction could also contribute to the pathogenesis of AF (25). The last but not the least, HT and AF might share the same pathogenic factors. For example, a recent MR study reported that higher BMI and a particularly fat mass index were associated with an increased risk of both HT and AF (26).

It was noted that in clinical practice, a retrospective real-world cohort analysis revealed that earlier and stricter 24-h blood pressure control reduced the occurrence of new-onset non-valvular AF (27). It was noted that HT might serve as a pathogeny for AF. Yet the current HT guidelines, including the recently released US guidelines (28), did not recommend more aggressive blood pressure targets for AF prevention. Based on epidemiological evidence, this suggestion might sound quite reasonable. The prevalence of HT increased and was currently ~20 to 50% in the adult population worldwide (29, 30). Our MR study revealed a causal association between HT and AF. So we believed that HT was still the most important potentially modifiable risk factor responsible for the increasing burden of AF. Although there was a genetic relationship between HT and AF, this relationship could not be explained by genetics alone. Many additional factors were relevant, including obesity, HF, sleep apnea, and so on. It should be further noted that genetics could not be impacted; other modifiable risk factors should be targeted to achieve better AF prevention.

## Limitations and Strengths

There are several limitations in our study. First, the ascertainment of AF is not perfect in the ARIC study. It is identified by ECGs performed during study exams, hospital discharge codes, and death certificates. So, it may miss the paroxysmal AF, resulting in a lower AF incidence. Second, technically speaking, it is better to adjust all the confounders, which may affect the AF incidence, such as rheumatic heart disease, chronic obstructive pulmonary disease, etc. Yet, we can only adjust as many confounders as we can get in the real world. Third, we suggest providing more omics information on the SNPs used in the analysis to see if these SNPs break the hypothesis of horizontal pleiotropy. The last but not the least, GWAS data in our study mainly relates to European ethnic individuals; therefore, the analysis should be repeated in other populations before being generalized across ethnic groups. In spite of this, based on the epidemiology data in ARIC study and Mendelian randomization causality research, we believe the causality to HT and AF.

## Conclusions

Participants with HT were associated with a 50% increased rate of incident AF. HT might be a genetic cause of AF.

## Data Availability Statement

The original contributions presented in the study are included in the article/Supplementary Material, further inquiries can be directed to the corresponding author/s.

## Ethics Statement

The studies involving human participants were reviewed and approved by Ethics Committee of Guangdong Pharmaceutical University. The participants provided written informed consent to participate in this study.

## Author Contributions

L-ZL: design of the study. X-YW: analysis the data. S-ZZ: interpretation of the data. W-DL: drafting of the article. X-DZ: conception and design of the study.

## Conflict of Interest

The authors declare that the research was conducted in the absence of any commercial or financial relationships that could be construed as a potential conflict of interest.

## Supplementary Material

Supplementary Figure 1 Diagram of the instrumental variable assumptions for Mendelian randomization. The three assumptions are: (1) the genetic variant must be robustly associated with the exposure; (2) the genetic variant should not be related to confounders of the exposure-outcome association; and (3) the genetic variant must influence the outcome through the exposure only and not through any direct or alternative pathways. The dashed lines represent pathways that violate the assumptions. AF, atrial fibrillation.

Supplementary Figure 2 Unadjusted cumulative curves for incident AF. During a median follow-up of 24.1 years, 1,414 cases (14.9%) of AF occurred. Unadjusted cumulative curves for incident AF are shown. AF, atrial fibrillation.

Supplementary Figure 3 Hazard ratio of SBP for AF. SBP, systolic blood pressure; AF, atrial fibrillation.

Supplementary Figure 4 Hazard ratio of DBP for AF. DBP, diastolic blood pressure; AF, atrial fibrillation.

Supplementary Figure 5 Subgroup and sensitivity analyses of the association between HT and incident AF. Pre-specified subgroups by sex, age, race, smoking, drinking, BMI, creatine, LDL-c, and TG were analyzed. Sensitivity analyses were conducted by excluding participants with prevalent HF, CHD, and diabetes. HT, hypertension; AF, atrial fibrillation, BMI, body mass index; LDL-c, low-density lipoprotein cholesterol; TG, triglycerides; HF, heart failure; CHD, coronary heart disease.

Supplementary Figure 6 Forest plots and scatter plots of causal effects between SDP-associated SNPs and risk of AF. The slopes of each line in the scatter plot represent the causal association for each method. SBP, systolic blood pressure; AF, atrial fibrillation; SNP, single-nucleotide polymorphism; MR, Mendelian randomization; IVW, inverse variance weighted.

Supplementary Figure 7 Forest plots and scatter plots of causal effects between DBP-associated SNPs and risk of AF. The slopes of each line in the scatter plot represent the causal association for each method. DBP, diastolic blood pressure; AF, atrial fibrillation; SNP, single-nucleotide polymorphism; MR, Mendelian randomization; IVW, inverse variance weighted.

Supplementary Figure 8 Leave-one-out sensitivity analysis and funnel plot in the SBP → AF MR analysis. **(A)** Le8ve-one-out sensitivity analysis. Each black point represents the IVW MR method applied to estimate the causal effect of SBP on AF, excluding that particular variant from the study. The red point depicts the IVW estimate using all of the SNPs. There are no instances where the exclusion of one particular SNP leads to dramatic changes in the overall result. **(B)** Funnel plot of the relationship between the causal effect of SBP on AF. Funnel plot showing the relationship between the causal effect of SBP on AF estimated using each SNP as a separate instrument against the inverse of the standard error of the causal estimate. Vertical lines show the causal estimates using all SNPs combined into a single instrument for the two different methods. Asymmetry in the funnel plot may be indicative of violations of the assumption through horizontal pleiotropy. SBP, systolic blood pressure; AF, atrial fibrillation; SNP, single-nucleotide polymorphism; MR, Mendelian randomization; IVW, inverse variance weighted.

Supplementary Figure 9 Leave-one-out sensitivity analysis and funnel plot in the DBP → AF MR analysis. **(A)** Leave-one-out sensitivity analysis. Each black point represents the IVW MR method applied to estimate the causal effect of DBP on AF, excluding that particular variant from the study. The red point depicts the IVW estimate using all of the SNPs. There are no instances where the exclusion of one particular SNP leads to dramatic changes in the overall result. **(B)** Funnel plot of the relationship between the causal effect of DBP on AF. Funnel plot showing the relationship between the causal effect of DBP on AF estimated using each SNP as a separate instrument against the inverse of the standard error of the causal estimate. Vertical lines show the causal estimates using all of the SNPs combined into a single instrument for the two different methods. Asymmetry in the funnel plot may be indicative of violations of the assumption through horizontal pleiotropy. DBP, diastolic blood pressure; AF, atrial fibrillation; SNP, single-nucleotide polymorphism; MR, Mendelian randomization; IVW, inverse variance weighted.

Supplementary Table 1 Characteristics of the SNPs associated with HT and AF.

Supplementary Table 2 Characteristics of the SNPs associated with SBP and AF.

Supplementary Table 3 Characteristics of the SNPs associated with DBP and AF.

Supplementary Table 4 Baseline characteristic of study populations by hypertension.

Supplementary Table 5 Hazard ratio of HT for AF.

Supplementary Table 6 Causal associations between genetically determined HT, SBP, DBP and AF.
