# Supplementary figures and images for: Hypertension and Atrial Fibrillation: A Study on Epidemiology and Mendelian Randomization Causality
Source: Front Cardiovasc Med. 2021 Mar 23;8:644405. doi: 10.3389/fcvm.2021.644405 (PMC8021766; doi:10.3389/fcvm.2021.644405)

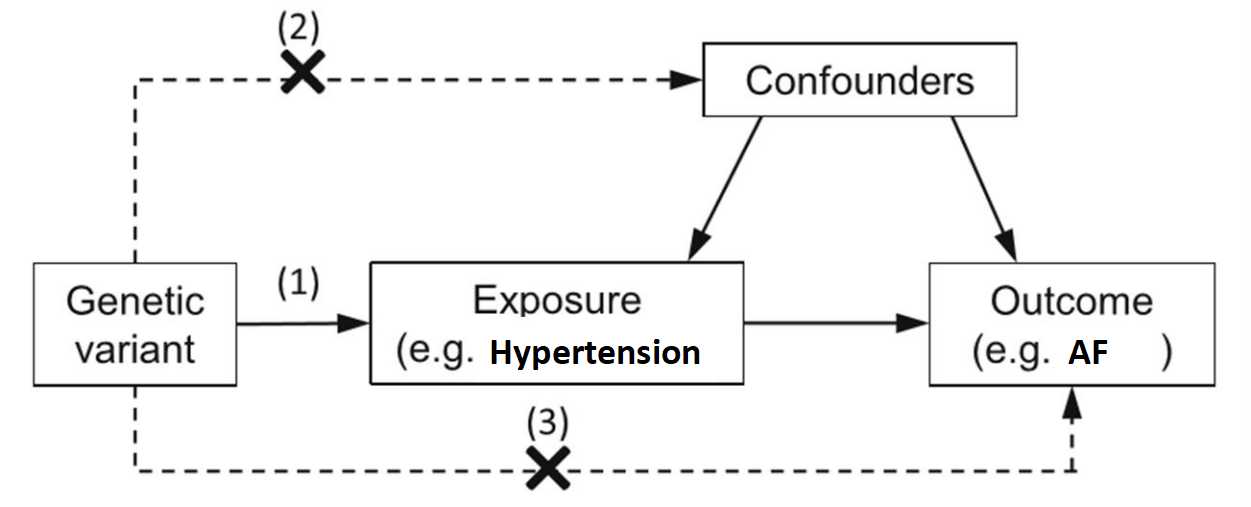

Supplement: Supplementary Figure 1 — Diagram of the instrumental variable assumptions for Mendelian randomization. The three assumptions are: (1) the genetic variant must be robustly associated with the exposure; (2) the genetic variant should not be related to confounders of the exposure-outcome association; and (3) the genetic variant must influence the outcome through the exposure only and not through any direct or alternative pathways. The dashed lines represent pathways that violate the assumptions. AF, atrial fibrillation. [file Image_1.jpg]

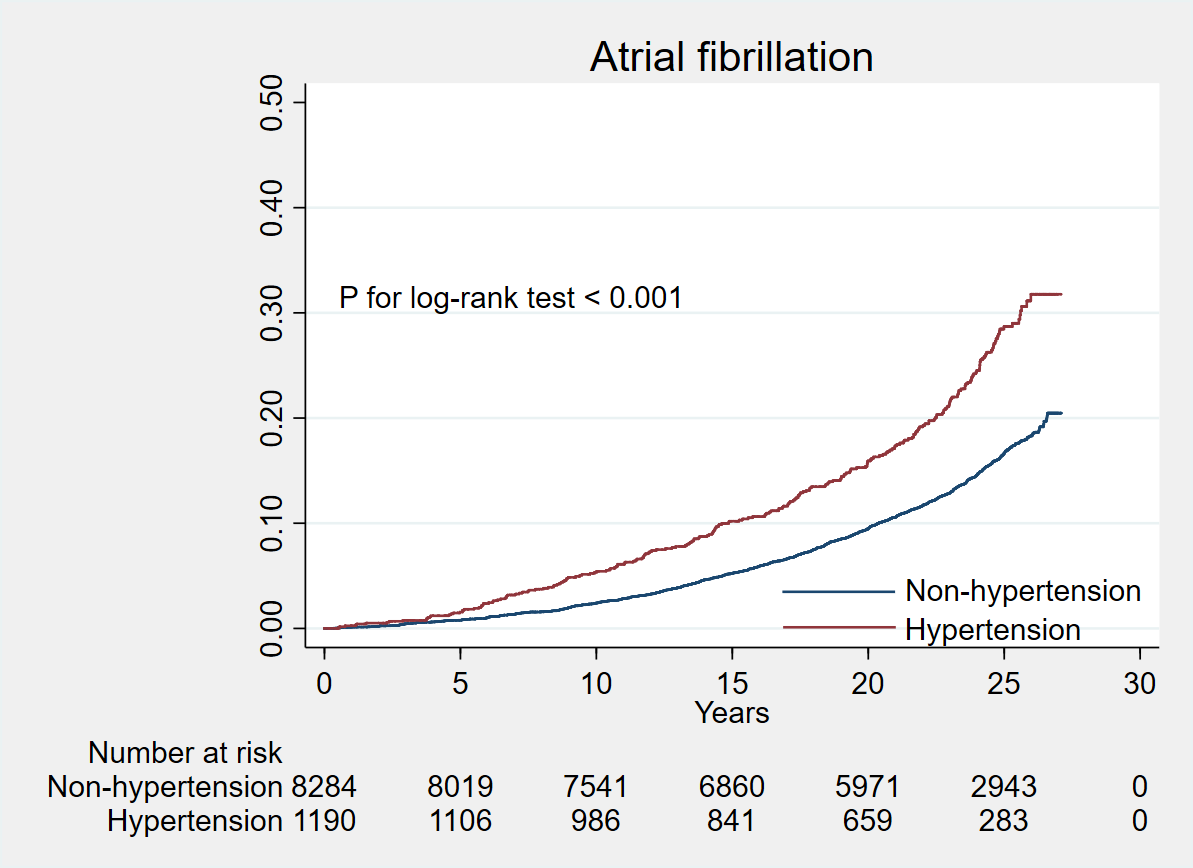

Supplement: Supplementary Figure 2 — Unadjusted cumulative curves for incident AF. During a median follow-up of 24.1 years, 1,414 cases (14.9%) of AF occurred. Unadjusted cumulative curves for incident AF are shown. AF, atrial fibrillation. [file Image_2.tif]

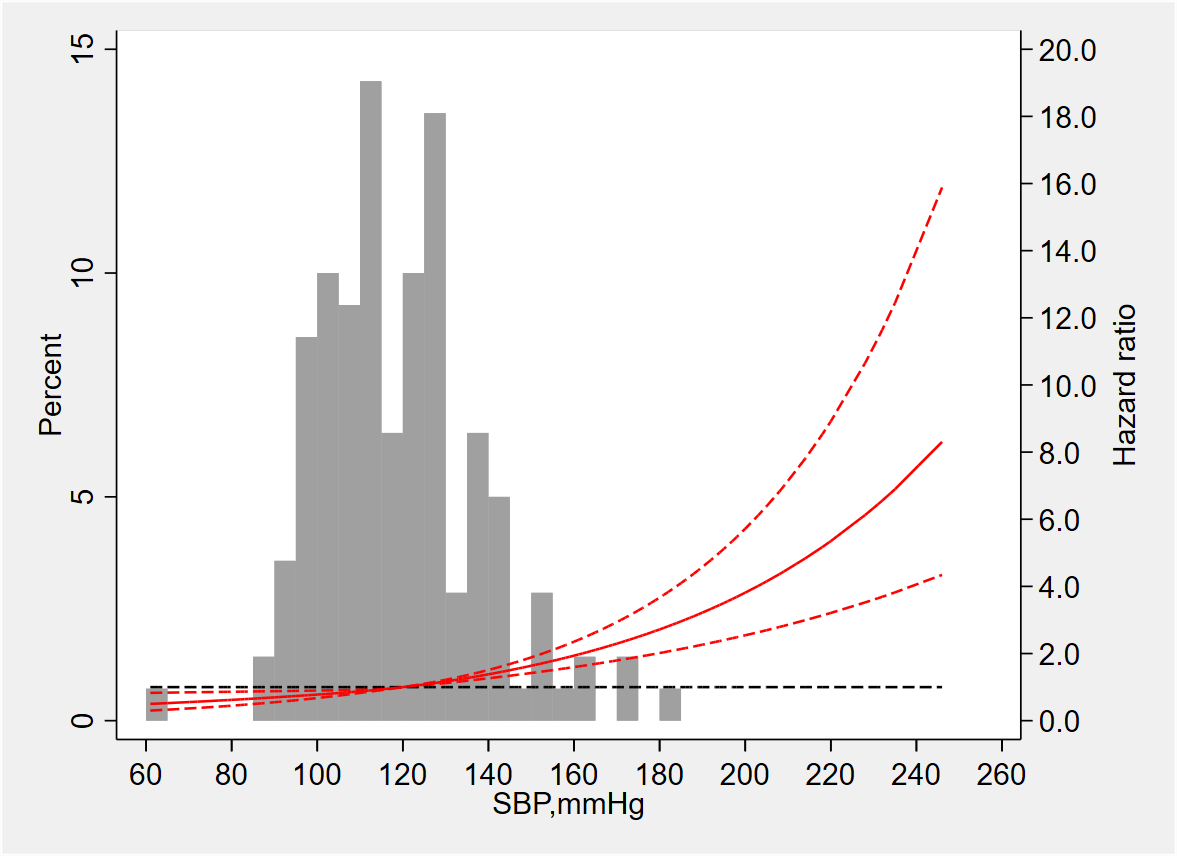

Supplement: Supplementary Figure 3 — Hazard ratio of SBP for AF. SBP, systolic blood pressure; AF, atrial fibrillation. [file Image_3.tif]

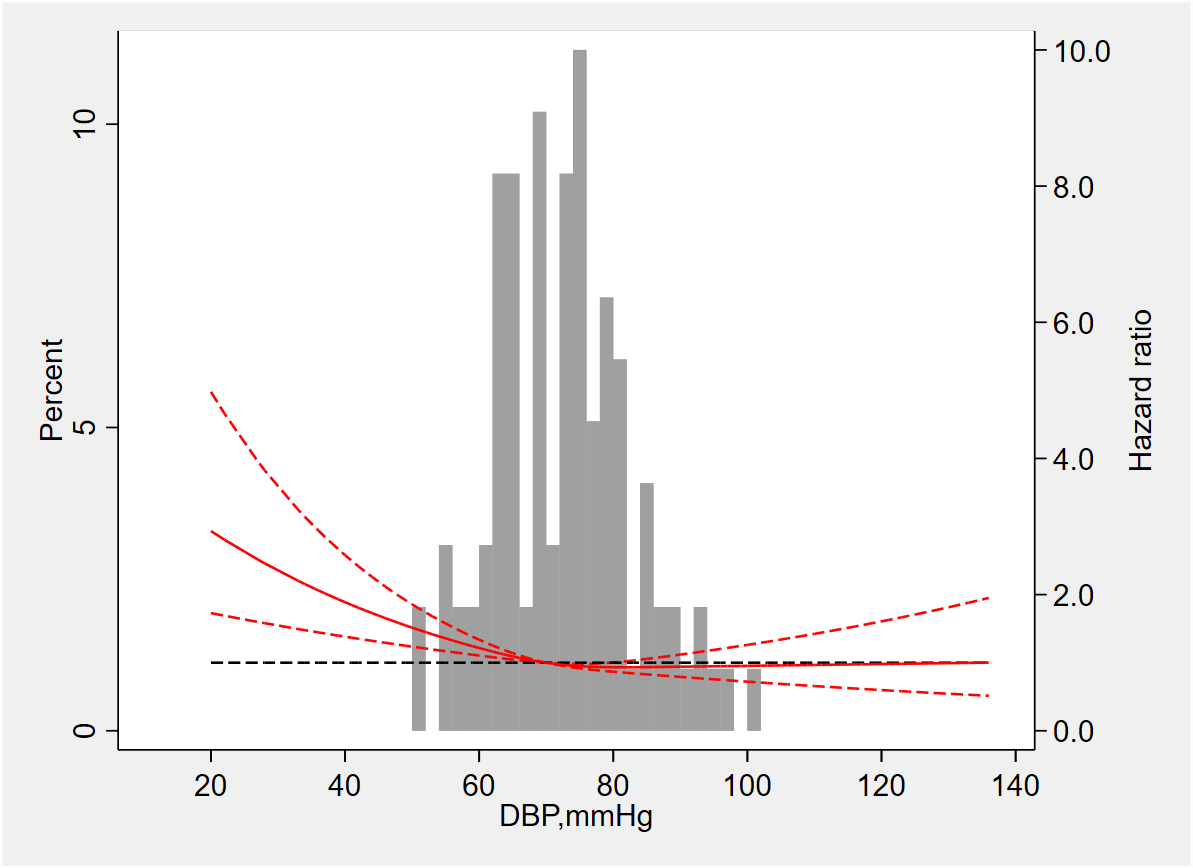

Supplement: Supplementary Figure 4 — Hazard ratio of DBP for AF. DBP, diastolic blood pressure; AF, atrial fibrillation. [file Image_4.tif]

A

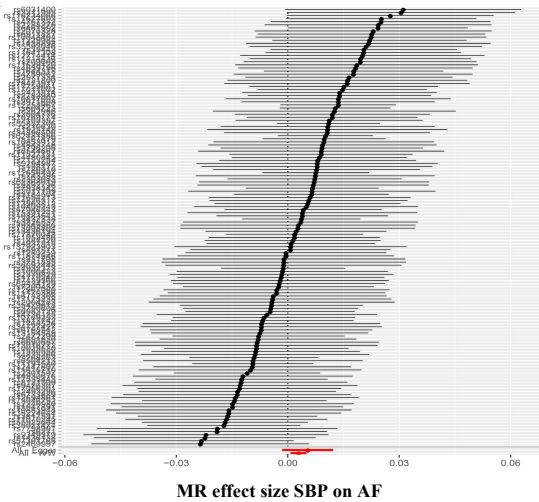

B

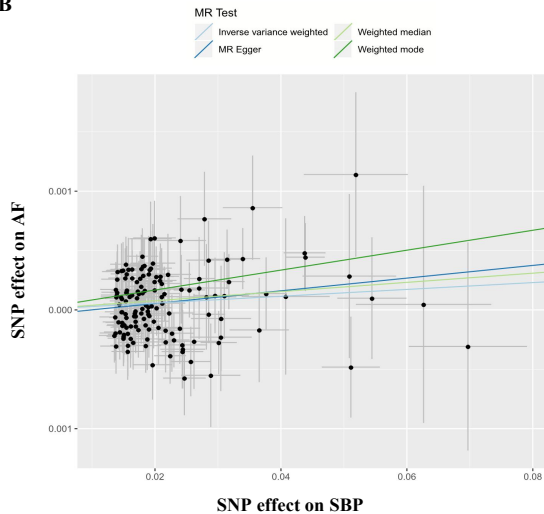

Supplement: Supplementary Figure 6 — Forest plots and scatter plots of causal effects between SDP-associated SNPs and risk of AF. The slopes of each line in the scatter plot represent the causal association for each method. SBP, systolic blood pressure; AF, atrial fibrillation; SNP, single-nucleotide polymorphism; MR, Mendelian randomization; IVW, inverse variance weighted. [file Image_6.pdf]

A

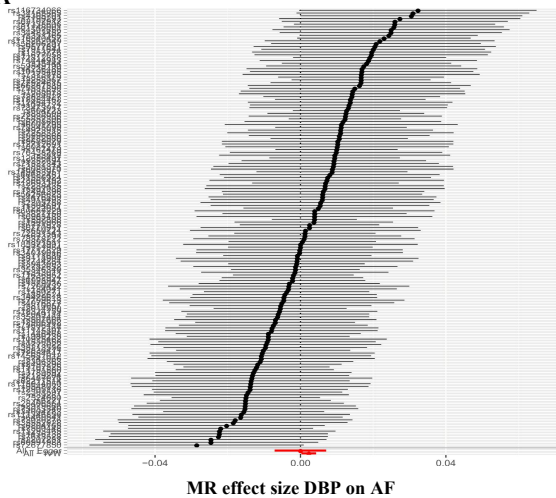

B

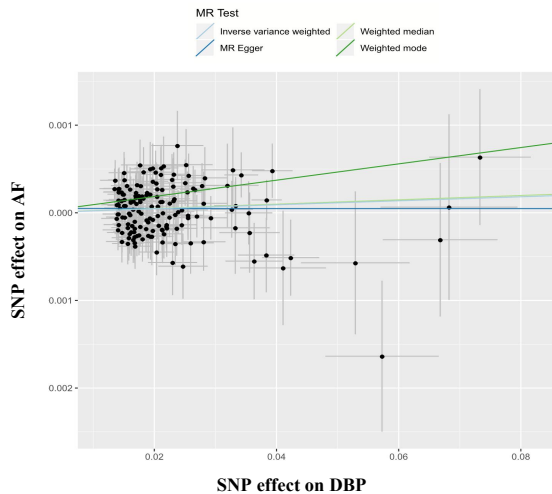

Supplement: Supplementary Figure 7 — Forest plots and scatter plots of causal effects between DBP-associated SNPs and risk of AF. The slopes of each line in the scatter plot represent the causal association for each method. DBP, diastolic blood pressure; AF, atrial fibrillation; SNP, single-nucleotide polymorphism; MR, Mendelian randomization; IVW, inverse variance weighted. [file Image_7.pdf]

**A**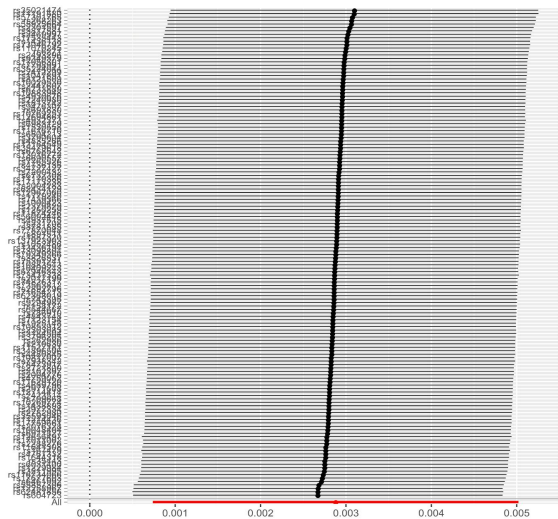

**MR leave-one-out sensitivity analysis for SBP on AF**

**B**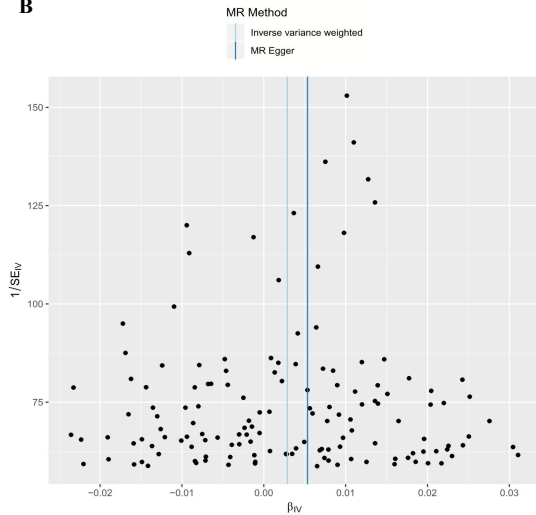

**Funnel plot**

Supplement: Supplementary Figure 8 — Leave-one-out sensitivity analysis and funnel plot in the SBP → AF MR analysis. (A) Le8ve-one-out sensitivity analysis. Each black point represents the IVW MR method applied to estimate the causal effect of SBP on AF, excluding that particular variant from the study. The red point depicts the IVW estimate using all of the SNPs. There are no instances where the exclusion of one particular SNP leads to dramatic changes in the overall result. (B) Funnel plot of the relationship between the causal effect of SBP on AF. Funnel plot showing the relationship between the causal effect of SBP on AF estimated using each SNP as a separate instrument against the inverse of the standard error of the causal estimate. Vertical lines show the causal estimates using all SNPs combined into a single instrument for the two different methods. Asymmetry in the funnel plot may be indicative of violations of the assumption through horizontal pleiotropy. SBP, systolic blood pressure; AF, atrial fibrillation; SNP, single-nucleotide polymorphism; MR, Mendelian randomization; IVW, inverse variance weighted. [file Image_8.pdf]

**A**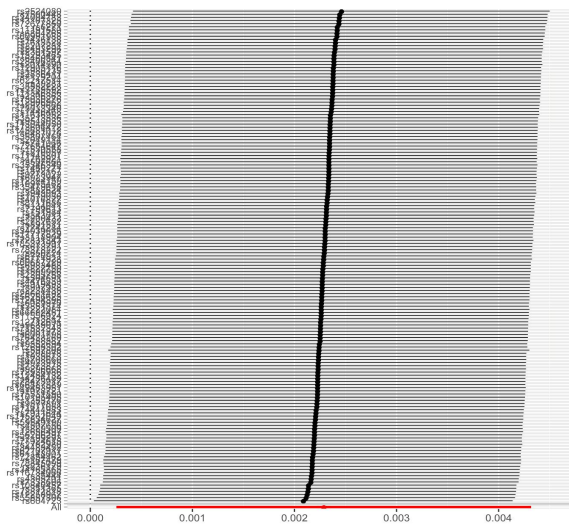

**MR leave-one-out sensitivity analysis for DBP on AF**

**B**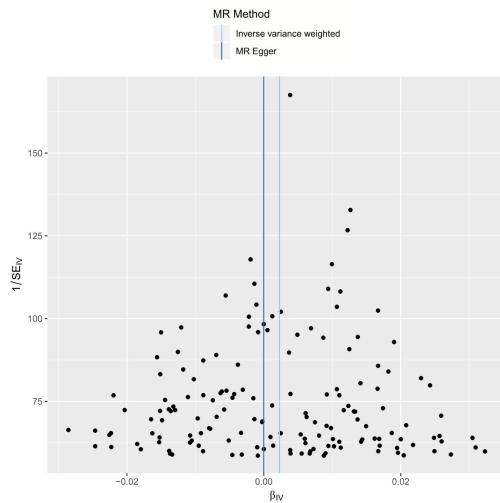

**Funnel plot**

Supplement: Supplementary Figure 9 — Leave-one-out sensitivity analysis and funnel plot in the DBP → AF MR analysis. (A) Leave-one-out sensitivity analysis. Each black point represents the IVW MR method applied to estimate the causal effect of DBP on AF, excluding that particular variant from the study. The red point depicts the IVW estimate using all of the SNPs. There are no instances where the exclusion of one particular SNP leads to dramatic changes in the overall result. (B) Funnel plot of the relationship between the causal effect of DBP on AF. Funnel plot showing the relationship between the causal effect of DBP on AF estimated using each SNP as a separate instrument against the inverse of the standard error of the causal estimate. Vertical lines show the causal estimates using all of the SNPs combined into a single instrument for the two different methods. Asymmetry in the funnel plot may be indicative of violations of the assumption through horizontal pleiotropy. DBP, diastolic blood pressure; AF, atrial fibrillation; SNP, single-nucleotide polymorphism; MR, Mendelian randomization; IVW, inverse variance weighted. [file Image_9.pdf]
