# Supplementary material for: Hypertension and Atrial Fibrillation: A Study on Epidemiology and Mendelian Randomization Causality
Source: Front Cardiovasc Med. 2021 Mar 23;8:644405. doi: 10.3389/fcvm.2021.644405 (PMC8021766; doi:10.3389/fcvm.2021.644405)

Subgroup	No. of Participants	No. of Events	Hazard ratio (95%CI)	P Value	P for interaction
Sex					0.237
Male	4494	806	1.42 (1.17, 1.73)	<0.001	
Female	4980	608	1.61 (1.29, 2.00)	<0.001	
Age, years					<0.001
<60	7932	1015	1.91 (1.61, 2.27)	<0.001	
≥60	1542	399	1.18 (0.91, 1.53)	0.211	
Race					0.930
Black	1830	187	1.51 (1.11, 2.06)	0.009	
White	7644	1227	1.50 (1.27, 1.76)	<0.001	
Smoking status					0.266
Current	2564	410	1.89 (1.44, 2.50)	<0.001	
Former	3084	510	1.42 (1.11, 1.80)	0.004	
Never	3826	494	1.37 (1.07, 1.74)	0.011	
Drinking status					0.649
Current	5802	874	1.47 (1.21, 1.78)	<0.001	
Former	1630	267	1.59 (1.13, 2.23)	0.007	
Never	2042	273	1.68 (1.24, 2.27)	0.001	
BMI,kg/m^2					0.993
<24	2817	331	1.49 (1.04, 2.13)	0.029	
24-28	3453	516	1.60 (1.24, 2.05)	<0.001	
≥28	3204	567	1.59 (1.29, 1.96)	<0.001	
Creatinine, mg/dl					0.264
<1.0	4378	581	1.74 (1.39, 2.18)	<0.001	
1.0-1.2	3435	547	1.34 (1.05, 1.70)	0.018	
≥1.2	1661	286	1.44 (1.06, 1.97)	0.021	
LDL-c, mmol/L					0.455
<3.5	4899	691	1.57 (1.28, 1.94)	<0.001	
≥3.5	4575	723	1.48 (1.21, 1.81)	<0.001	
TG, mmol/L					0.394
<1.7	7349	1062	1.52 (1.28, 1.81)	<0.001	
≥1.7	2125	352	1.50 (1.14, 1.97)	0.004	
Sensitivity analyse					
Excluded of participants with HF	9459	1404	1.50 (1.30, 1.73)	<0.001	
Excluded of participants with CHD	9269	1357	1.49 (1.29, 1.73)	<0.001	
Excluded of participants with Diabetes	8926	1299	1.53 (1.31, 1.78)	<0.001	

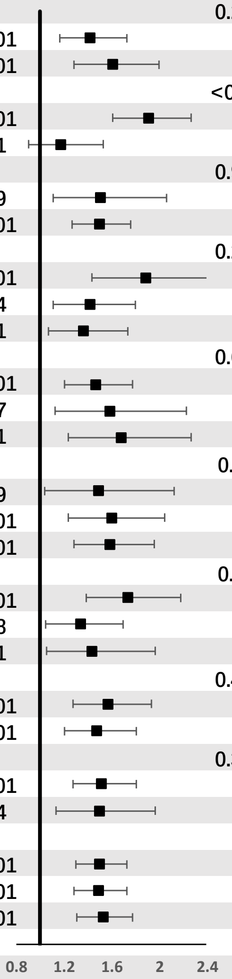

Supplement: Supplementary Figure 5 — Subgroup and sensitivity analyses of the association between HT and incident AF. Pre-specified subgroups by sex, age, race, smoking, drinking, BMI, creatine, LDL-c, and TG were analyzed. Sensitivity analyses were conducted by excluding participants with prevalent HF, CHD, and diabetes. HT, hypertension; AF, atrial fibrillation, BMI, body mass index; LDL-c, low-density lipoprotein cholesterol; TG, triglycerides; HF, heart failure; CHD, coronary heart disease. [file Image_5.pdf]
