# Supplementary material for: Hypertension and Atrial Fibrillation: A Study on Epidemiology and Mendelian Randomization Causality
Source: Front Cardiovasc Med. 2021 Mar 23;8:644405. doi: 10.3389/fcvm.2021.644405 (PMC8021766; doi:10.3389/fcvm.2021.644405)
Supplement: Supplementary Table 1 — Characteristics of the SNPs associated with HT and AF. [file Table_1.docx]

**Table** **Supplement 1 Characteristics of the SNPs associated with HT and AF**

| SNP | EA | Other | EAF | Associations with HT | | |  | Associations with AF | | | Management |
| --- | --- | --- | --- | --- | --- | --- | --- | --- | --- | --- | --- |
|  |  | allele |  | Beta | se | P value |  | Beta | se | P value |  |
| rs141878088 | T | C | 0.023 | 0.002 | 0.000 | 0.000 |  | 0.002 | 0.001 | 0.034 | include |
| rs144425491 | A | G | 0.007 | 0.004 | 0.001 | 0.000 |  | 0.002 | 0.001 | 0.200 |  |
| rs75558191 | T | C | 0.135 | 0.001 | 0.000 | 0.000 |  | 0.000 | 0.000 | 0.210 |  |

HT, Hypertension; AF, Atrial fibrillation; EA, Effect allele; EAF, Effect allele frequency; se, Standard error; SNP, Single-nucleotide polymorphism.
