# Supplementary material for: Hypertension and Atrial Fibrillation: A Study on Epidemiology and Mendelian Randomization Causality
Source: Front Cardiovasc Med. 2021 Mar 23;8:644405. doi: 10.3389/fcvm.2021.644405 (PMC8021766; doi:10.3389/fcvm.2021.644405)
Supplement: Supplementary Table 2 — Characteristics of the SNPs associated with SBP and AF. [file Table_2.docx]

**Table Supplement 2 Characteristics of the SNPs associated with SBP and AF**

| SNP | EA | Other | EAF | Associations with SBP | | |  | Associations with AF | | | Management |
| --- | --- | --- | --- | --- | --- | --- | --- | --- | --- | --- | --- |
|  |  | allele |  | Beta | se | P value |  | Beta | se | P value |  |
| rs365990 | G | A | 0.369 | -0.017 | 0.003 | 0.000 |  | 0.001 | 0.000 | 0.000 | Exclude* |
| rs2249105 | G | A | 0.361 | -0.018 | 0.003 | 0.000 |  | -0.001 | 0.000 | 0.000 |  |
| rs78744936 | A | G | 0.269 | 0.016 | 0.003 | 0.000 |  | -0.001 | 0.000 | 0.000 |  |
| rs2306526 | T | C | 0.525 | -0.016 | 0.002 | 0.000 |  | -0.001 | 0.000 | 0.000 |  |
| rs11874 | A | G | 0.137 | 0.027 | 0.004 | 0.000 |  | 0.001 | 0.000 | 0.001 |  |
| rs9888615 | C | T | 0.711 | 0.017 | 0.003 | 0.000 |  | -0.001 | 0.000 | 0.002 |  |
| rs2627316 | G | A | 0.471 | 0.019 | 0.002 | 0.000 |  | 0.001 | 0.000 | 0.003 |  |
| rs10857147 | T | A | 0.292 | 0.037 | 0.003 | 0.000 |  | 0.001 | 0.000 | 0.005 |  |
| rs6026742 | A | G | 0.118 | 0.032 | 0.004 | 0.000 |  | 0.001 | 0.000 | 0.006 |  |
| rs2052691 | A | G | 0.288 | 0.019 | 0.003 | 0.000 |  | 0.001 | 0.000 | 0.010 |  |
| rs55938136 | G | A | 0.226 | -0.017 | 0.003 | 0.000 |  | 0.001 | 0.000 | 0.010 |  |
| rs60691990 | C | T | 0.343 | -0.020 | 0.003 | 0.000 |  | 0.001 | 0.000 | 0.011 |  |
| rs2643826 | T | C | 0.453 | 0.016 | 0.002 | 0.000 |  | 0.001 | 0.000 | 0.012 |  |
| rs2177843 | T | C | 0.146 | 0.024 | 0.003 | 0.000 |  | -0.001 | 0.000 | 0.014 |  |
| rs138110118 | G | T | 0.114 | -0.024 | 0.004 | 0.000 |  | 0.001 | 0.000 | 0.017 |  |
| rs144356415 | G | A | 0.040 | 0.036 | 0.006 | 0.000 |  | -0.001 | 0.001 | 0.023 |  |
| rs79621605 | C | T | 0.034 | -0.037 | 0.007 | 0.000 |  | 0.001 | 0.001 | 0.027 |  |
| rs488834 | T | C | 0.767 | -0.017 | 0.003 | 0.000 |  | -0.001 | 0.000 | 0.032 |  |
| rs75989961 | G | T | 0.082 | 0.025 | 0.004 | 0.000 |  | 0.001 | 0.000 | 0.032 |  |
| rs28667801 | T | A | 0.406 | 0.014 | 0.003 | 0.000 |  | 0.000 | 0.000 | 0.035 |  |
| rs1543270 | T | C | 0.455 | -0.016 | 0.002 | 0.000 |  | 0.000 | 0.000 | 0.036 |  |
| rs6734118 | A | C | 0.216 | -0.019 | 0.003 | 0.000 |  | -0.001 | 0.000 | 0.039 |  |
| rs6461992 | G | A | 0.927 | 0.036 | 0.005 | 0.000 |  | 0.001 | 0.000 | 0.050 | include |
| rs3211995 | A | G | 0.159 | -0.020 | 0.003 | 0.000 |  | -0.001 | 0.000 | 0.052 |  |
| rs116734066 | T | C | 0.093 | -0.028 | 0.004 | 0.000 |  | -0.001 | 0.000 | 0.053 |  |
| rs17677603 | G | A | 0.395 | 0.018 | 0.003 | 0.000 |  | 0.000 | 0.000 | 0.054 |  |
| rs6031400 | T | C | 0.160 | 0.019 | 0.003 | 0.000 |  | 0.001 | 0.000 | 0.055 |  |
| rs57301765 | A | G | 0.154 | 0.025 | 0.003 | 0.000 |  | -0.001 | 0.000 | 0.067 |  |
| rs141878088 | T | C | 0.023 | 0.002 | 0.000 | 0.000 |  | 0.002 | 0.001 | 0.081 |  |
| rs12258967 | G | C | 0.298 | -0.031 | 0.003 | 0.000 |  | 0.000 | 0.000 | 0.087 |  |
| rs604723 | C | T | 0.724 | 0.034 | 0.003 | 0.000 |  | 0.000 | 0.000 | 0.093 |  |
| rs4767332 | A | C | 0.581 | 0.015 | 0.002 | 0.000 |  | 0.000 | 0.000 | 0.096 |  |
| rs1229984 | C | T | 0.978 | 0.052 | 0.008 | 0.000 |  | 0.001 | 0.001 | 0.100 |  |
| rs35021474 | G | C | 0.617 | -0.022 | 0.003 | 0.000 |  | 0.000 | 0.000 | 0.100 |  |
| rs35443 | C | G | 0.383 | -0.018 | 0.003 | 0.000 |  | 0.000 | 0.000 | 0.110 |  |
| rs2288276 | C | G | 0.897 | 0.024 | 0.004 | 0.000 |  | 0.001 | 0.000 | 0.120 |  |
| rs2469997 | C | G | 0.815 | -0.020 | 0.003 | 0.000 |  | 0.000 | 0.000 | 0.120 |  |
| rs55857306 | A | G | 0.164 | -0.044 | 0.003 | 0.000 |  | 0.000 | 0.000 | 0.120 |  |
| rs62481856 | A | G | 0.197 | 0.044 | 0.003 | 0.000 |  | 0.000 | 0.000 | 0.120 |  |
| rs7753358 | A | T | 0.487 | 0.014 | 0.003 | 0.000 |  | 0.000 | 0.000 | 0.120 |  |
| rs11641308 | C | T | 0.653 | 0.018 | 0.003 | 0.000 |  | 0.000 | 0.000 | 0.130 |  |
| rs1436138 | G | A | 0.358 | -0.016 | 0.003 | 0.000 |  | 0.000 | 0.000 | 0.140 |  |
| rs55925664 | A | T | 0.186 | 0.026 | 0.003 | 0.000 |  | 0.000 | 0.000 | 0.140 |  |
| rs1644318 | C | T | 0.385 | 0.019 | 0.003 | 0.000 |  | 0.000 | 0.000 | 0.150 |  |
| rs6911827 | T | C | 0.454 | 0.015 | 0.002 | 0.000 |  | 0.000 | 0.000 | 0.150 |  |
| rs10048404 | T | C | 0.367 | -0.015 | 0.003 | 0.000 |  | 0.000 | 0.000 | 0.160 |  |
| rs2076328 | T | G | 0.500 | -0.014 | 0.002 | 0.000 |  | 0.000 | 0.000 | 0.160 |  |
| rs146408037 | A | C | 0.002 | 0.006 | 0.001 | 0.000 |  | -0.004 | 0.003 | 0.160 |  |
| rs35593046 | T | G | 0.270 | -0.016 | 0.003 | 0.000 |  | 0.000 | 0.000 | 0.190 |  |
| rs3817581 | T | C | 0.493 | 0.019 | 0.002 | 0.000 |  | 0.000 | 0.000 | 0.190 |  |
| rs9349379 | G | A | 0.405 | -0.014 | 0.003 | 0.000 |  | 0.000 | 0.000 | 0.190 |  |
| rs11214436 | T | G | 0.382 | -0.015 | 0.003 | 0.000 |  | 0.000 | 0.000 | 0.200 |  |
| rs1438898 | C | A | 0.251 | 0.016 | 0.003 | 0.000 |  | 0.000 | 0.000 | 0.200 |  |
| rs17011002 | G | C | 0.141 | 0.029 | 0.004 | 0.000 |  | 0.000 | 0.000 | 0.210 |  |
| rs6271 | T | C | 0.074 | -0.029 | 0.005 | 0.000 |  | 0.001 | 0.000 | 0.210 |  |
| rs709668 | G | A | 0.798 | 0.018 | 0.003 | 0.000 |  | 0.000 | 0.000 | 0.220 |  |
| rs11070245 | G | T | 0.530 | 0.017 | 0.002 | 0.000 |  | 0.000 | 0.000 | 0.230 |  |
| rs12714414 | C | T | 0.160 | -0.020 | 0.004 | 0.000 |  | 0.000 | 0.000 | 0.230 |  |
| rs17759661 | A | C | 0.498 | 0.018 | 0.002 | 0.000 |  | 0.000 | 0.000 | 0.240 |  |
| rs167479 | T | G | 0.470 | -0.027 | 0.002 | 0.000 |  | 0.000 | 0.000 | 0.250 |  |
| rs2977334 | T | G | 0.595 | 0.016 | 0.003 | 0.000 |  | 0.000 | 0.000 | 0.250 |  |
| rs7798991 | C | T | 0.262 | 0.016 | 0.003 | 0.000 |  | 0.000 | 0.000 | 0.250 |  |
| rs11191580 | C | T | 0.077 | -0.051 | 0.005 | 0.000 |  | 0.000 | 0.000 | 0.260 |  |
| rs4639796 | A | G | 0.159 | 0.019 | 0.003 | 0.000 |  | 0.000 | 0.000 | 0.260 |  |
| rs73046792 | A | G | 0.165 | -0.024 | 0.003 | 0.000 |  | 0.000 | 0.000 | 0.260 |  |
| rs11629120 | C | T | 0.414 | 0.014 | 0.003 | 0.000 |  | 0.000 | 0.000 | 0.270 |  |
| rs2301597 | C | T | 0.576 | -0.023 | 0.002 | 0.000 |  | 0.000 | 0.000 | 0.270 |  |
| rs2782980 | C | T | 0.722 | 0.020 | 0.003 | 0.000 |  | 0.000 | 0.000 | 0.270 |  |
| rs4759062 | T | C | 0.298 | -0.015 | 0.003 | 0.000 |  | 0.000 | 0.000 | 0.280 |  |
| rs144425491 | A | G | 0.007 | 0.004 | 0.001 | 0.000 |  | 0.002 | 0.002 | 0.289 |  |
| rs17535443 | A | G | 0.277 | -0.022 | 0.003 | 0.000 |  | 0.000 | 0.000 | 0.290 |  |
| rs2423514 | G | A | 0.463 | -0.017 | 0.002 | 0.000 |  | 0.000 | 0.000 | 0.300 |  |
| rs268263 | A | T | 0.754 | 0.030 | 0.003 | 0.000 |  | 0.000 | 0.000 | 0.300 |  |
| rs35224044 | T | C | 0.585 | 0.015 | 0.002 | 0.000 |  | 0.000 | 0.000 | 0.300 |  |
| rs10269774 | A | G | 0.324 | -0.021 | 0.003 | 0.000 |  | 0.000 | 0.000 | 0.310 |  |
| rs12656497 | C | T | 0.596 | 0.032 | 0.003 | 0.000 |  | 0.000 | 0.000 | 0.310 |  |
| rs2971608 | C | T | 0.221 | 0.021 | 0.003 | 0.000 |  | 0.000 | 0.000 | 0.310 |  |
| rs2493296 | T | C | 0.137 | 0.024 | 0.004 | 0.000 |  | 0.000 | 0.000 | 0.320 |  |
| rs1047891 | A | C | 0.316 | -0.016 | 0.003 | 0.000 |  | 0.000 | 0.000 | 0.330 |  |
| rs2721800 | C | G | 0.181 | -0.018 | 0.003 | 0.000 |  | 0.000 | 0.000 | 0.330 |  |
| rs78473917 | C | T | 0.149 | -0.019 | 0.003 | 0.000 |  | 0.000 | 0.000 | 0.340 |  |
| rs10883948 | T | G | 0.504 | -0.014 | 0.002 | 0.000 |  | 0.000 | 0.000 | 0.350 |  |
| rs17056301 | C | T | 0.254 | 0.019 | 0.003 | 0.000 |  | 0.000 | 0.000 | 0.350 |  |
| rs2004776 | T | C | 0.240 | 0.020 | 0.003 | 0.000 |  | 0.000 | 0.000 | 0.370 |  |
| rs2240980 | G | C | 0.293 | 0.015 | 0.003 | 0.000 |  | 0.000 | 0.000 | 0.370 |  |
| rs10817007 | G | T | 0.128 | 0.022 | 0.004 | 0.000 |  | 0.000 | 0.000 | 0.380 |  |
| rs6733889 | C | T | 0.623 | -0.015 | 0.003 | 0.000 |  | 0.000 | 0.000 | 0.380 |  |
| rs2102397 | C | A | 0.499 | -0.018 | 0.002 | 0.000 |  | 0.000 | 0.000 | 0.390 |  |
| rs2274224 | C | G | 0.432 | -0.025 | 0.002 | 0.000 |  | 0.000 | 0.000 | 0.390 |  |
| rs8121509 | C | T | 0.452 | -0.016 | 0.002 | 0.000 |  | 0.000 | 0.000 | 0.390 |  |
| rs12694861 | A | G | 0.605 | 0.014 | 0.003 | 0.000 |  | 0.000 | 0.000 | 0.400 |  |
| rs4930676 | T | C | 0.108 | -0.024 | 0.004 | 0.000 |  | 0.000 | 0.000 | 0.420 |  |
| rs9476307 | G | A | 0.411 | -0.014 | 0.003 | 0.000 |  | 0.000 | 0.000 | 0.430 |  |
| rs262986 | A | G | 0.468 | -0.014 | 0.002 | 0.000 |  | 0.000 | 0.000 | 0.450 |  |
| rs34896506 | C | T | 0.177 | 0.021 | 0.003 | 0.000 |  | 0.000 | 0.000 | 0.450 |  |
| rs210630 | G | A | 0.501 | -0.016 | 0.002 | 0.000 |  | 0.000 | 0.000 | 0.470 |  |
| rs7938342 | A | T | 0.584 | 0.025 | 0.003 | 0.000 |  | 0.000 | 0.000 | 0.470 |  |
| rs11527181 | G | A | 0.434 | -0.018 | 0.002 | 0.000 |  | 0.000 | 0.000 | 0.480 |  |
| rs2447607 | T | C | 0.618 | 0.017 | 0.003 | 0.000 |  | 0.000 | 0.000 | 0.480 |  |
| rs4480845 | C | T | 0.640 | -0.020 | 0.003 | 0.000 |  | 0.000 | 0.000 | 0.480 |  |
| rs2472299 | G | A | 0.728 | -0.022 | 0.003 | 0.000 |  | 0.000 | 0.000 | 0.500 |  |
| rs10029530 | T | A | 0.659 | -0.019 | 0.003 | 0.000 |  | 0.000 | 0.000 | 0.510 |  |
| rs11616710 | T | C | 0.101 | 0.026 | 0.004 | 0.000 |  | 0.000 | 0.000 | 0.510 |  |
| rs3796205 | C | G | 0.351 | -0.017 | 0.003 | 0.000 |  | 0.000 | 0.000 | 0.510 |  |
| rs10853912 | T | C | 0.399 | 0.015 | 0.003 | 0.000 |  | 0.000 | 0.000 | 0.520 |  |
| rs1925148 | G | A | 0.560 | -0.014 | 0.002 | 0.000 |  | 0.000 | 0.000 | 0.520 |  |
| rs6504213 | C | T | 0.592 | 0.015 | 0.003 | 0.000 |  | 0.000 | 0.000 | 0.530 |  |
| rs75558191 | T | C | 0.135 | 0.001 | 0.000 | 0.000 |  | 0.000 | 0.000 | 0.550 |  |
| rs1530558 | C | T | 0.136 | -0.023 | 0.004 | 0.000 |  | 0.000 | 0.000 | 0.550 |  |
| rs2303083 | A | G | 0.195 | -0.024 | 0.003 | 0.000 |  | 0.000 | 0.000 | 0.550 |  |
| rs3184504 | C | T | 0.518 | -0.021 | 0.002 | 0.000 |  | 0.000 | 0.000 | 0.550 |  |
| rs4843748 | A | G | 0.669 | -0.015 | 0.003 | 0.000 |  | 0.000 | 0.000 | 0.550 |  |
| rs691830 | G | A | 0.477 | 0.017 | 0.002 | 0.000 |  | 0.000 | 0.000 | 0.550 |  |
| rs7123754 | C | T | 0.327 | -0.018 | 0.003 | 0.000 |  | 0.000 | 0.000 | 0.550 |  |
| rs4835266 | C | T | 0.484 | -0.015 | 0.002 | 0.000 |  | 0.000 | 0.000 | 0.570 |  |
| rs1813742 | T | C | 0.588 | 0.018 | 0.002 | 0.000 |  | 0.000 | 0.000 | 0.590 |  |
| rs6544667 | T | C | 0.383 | -0.014 | 0.003 | 0.000 |  | 0.000 | 0.000 | 0.590 |  |
| rs786910 | G | C | 0.589 | -0.016 | 0.002 | 0.000 |  | 0.000 | 0.000 | 0.590 |  |
| rs10769253 | A | G | 0.177 | -0.024 | 0.003 | 0.000 |  | 0.000 | 0.000 | 0.610 |  |
| rs13016772 | T | C | 0.762 | 0.016 | 0.003 | 0.000 |  | 0.000 | 0.000 | 0.610 |  |
| rs13154549 | G | A | 0.068 | 0.030 | 0.005 | 0.000 |  | 0.000 | 0.000 | 0.610 |  |
| rs158172 | G | A | 0.219 | -0.017 | 0.003 | 0.000 |  | 0.000 | 0.000 | 0.620 |  |
| rs6690557 | G | T | 0.284 | -0.015 | 0.003 | 0.000 |  | 0.000 | 0.000 | 0.620 |  |
| rs9294987 | C | T | 0.502 | 0.014 | 0.002 | 0.000 |  | 0.000 | 0.000 | 0.630 |  |
| rs6768542 | A | G | 0.158 | -0.021 | 0.003 | 0.000 |  | 0.000 | 0.000 | 0.640 |  |
| rs73437338 | C | T | 0.167 | -0.038 | 0.003 | 0.000 |  | 0.000 | 0.000 | 0.650 |  |
| rs743395 | T | C | 0.375 | 0.015 | 0.003 | 0.000 |  | 0.000 | 0.000 | 0.650 |  |
| rs62368019 | C | T | 0.290 | 0.016 | 0.003 | 0.000 |  | 0.000 | 0.000 | 0.660 |  |
| rs7765526 | G | A | 0.542 | -0.014 | 0.002 | 0.000 |  | 0.000 | 0.000 | 0.660 |  |
| rs2892796 | A | G | 0.078 | -0.027 | 0.005 | 0.000 |  | 0.000 | 0.000 | 0.670 |  |
| rs34727427 | C | T | 0.319 | 0.015 | 0.003 | 0.000 |  | 0.000 | 0.000 | 0.670 |  |
| rs71654213 | T | C | 0.385 | -0.017 | 0.003 | 0.000 |  | 0.000 | 0.000 | 0.670 |  |
| rs6983129 | A | C | 0.522 | -0.020 | 0.002 | 0.000 |  | 0.000 | 0.000 | 0.680 |  |
| rs73563812 | T | G | 0.236 | -0.021 | 0.003 | 0.000 |  | 0.000 | 0.000 | 0.680 |  |
| rs74826317 | G | A | 0.028 | -0.051 | 0.007 | 0.000 |  | 0.000 | 0.001 | 0.680 |  |
| rs2017199 | A | G | 0.301 | -0.015 | 0.003 | 0.000 |  | 0.000 | 0.000 | 0.700 |  |
| rs3790604 | A | C | 0.074 | 0.037 | 0.005 | 0.000 |  | 0.000 | 0.000 | 0.700 |  |
| rs57946343 | C | T | 0.152 | -0.030 | 0.003 | 0.000 |  | 0.000 | 0.000 | 0.700 |  |
| rs4736135 | T | C | 0.714 | -0.017 | 0.003 | 0.000 |  | 0.000 | 0.000 | 0.710 |  |
| rs35479618 | A | G | 0.017 | 0.070 | 0.009 | 0.000 |  | 0.000 | 0.001 | 0.730 |  |
| rs9385405 | C | G | 0.436 | 0.023 | 0.002 | 0.000 |  | 0.000 | 0.000 | 0.730 |  |
| rs10883543 | T | G | 0.888 | 0.031 | 0.004 | 0.000 |  | 0.000 | 0.000 | 0.740 |  |
| rs10409243 | T | C | 0.602 | -0.015 | 0.003 | 0.000 |  | 0.000 | 0.000 | 0.750 |  |
| rs17173238 | G | A | 0.286 | 0.015 | 0.003 | 0.000 |  | 0.000 | 0.000 | 0.800 |  |
| rs2004283 | G | T | 0.578 | -0.014 | 0.002 | 0.000 |  | 0.000 | 0.000 | 0.800 |  |
| rs6732308 | G | A | 0.208 | -0.018 | 0.003 | 0.000 |  | 0.000 | 0.000 | 0.800 |  |
| rs75391241 | G | A | 0.072 | 0.028 | 0.005 | 0.000 |  | 0.000 | 0.000 | 0.800 |  |
| rs3826537 | G | A | 0.428 | 0.014 | 0.002 | 0.000 |  | 0.000 | 0.000 | 0.830 |  |
| rs11170386 | T | C | 0.288 | -0.017 | 0.003 | 0.000 |  | 0.000 | 0.000 | 0.840 |  |
| rs12967060 | T | C | 0.267 | 0.017 | 0.003 | 0.000 |  | 0.000 | 0.000 | 0.850 |  |
| rs7200432 | A | G | 0.300 | -0.019 | 0.003 | 0.000 |  | 0.000 | 0.000 | 0.850 |  |
| rs77870048 | T | C | 0.053 | 0.054 | 0.005 | 0.000 |  | 0.000 | 0.001 | 0.850 |  |
| rs13436194 | G | A | 0.426 | -0.019 | 0.002 | 0.000 |  | 0.000 | 0.000 | 0.860 |  |
| rs79349366 | T | C | 0.033 | -0.041 | 0.007 | 0.000 |  | 0.000 | 0.001 | 0.860 |  |
| rs62434129 | T | A | 0.071 | -0.030 | 0.005 | 0.000 |  | 0.000 | 0.000 | 0.870 |  |
| rs73098804 | A | T | 0.130 | 0.021 | 0.004 | 0.000 |  | 0.000 | 0.000 | 0.870 |  |
| rs1052486 | G | A | 0.475 | 0.019 | 0.002 | 0.000 |  | 0.000 | 0.000 | 0.880 |  |
| rs4932373 | C | A | 0.326 | 0.029 | 0.003 | 0.000 |  | 0.000 | 0.000 | 0.880 |  |
| rs7439366 | C | T | 0.455 | 0.015 | 0.002 | 0.000 |  | 0.000 | 0.000 | 0.890 |  |
| rs1779240 | A | G | 0.764 | -0.019 | 0.003 | 0.000 |  | 0.000 | 0.000 | 0.900 |  |
| rs1887320 | A | G | 0.477 | 0.019 | 0.002 | 0.000 |  | 0.000 | 0.000 | 0.910 |  |
| rs1000423 | T | C | 0.735 | 0.018 | 0.003 | 0.000 |  | 0.000 | 0.000 | 0.920 |  |
| rs2379829 | C | G | 0.733 | -0.017 | 0.003 | 0.000 |  | 0.000 | 0.000 | 0.920 |  |
| rs4937515 | C | G | 0.595 | -0.020 | 0.003 | 0.000 |  | 0.000 | 0.000 | 0.940 |  |
| rs56092448 | T | C | 0.114 | 0.022 | 0.004 | 0.000 |  | 0.000 | 0.000 | 0.940 |  |
| rs231708 | C | G | 0.687 | -0.015 | 0.003 | 0.000 |  | 0.000 | 0.000 | 0.950 |  |
| rs3781885 | T | C | 0.313 | -0.015 | 0.003 | 0.000 |  | 0.000 | 0.000 | 0.950 |  |
| rs137923903 | T | C | 0.013 | -0.063 | 0.011 | 0.000 |  | 0.000 | 0.001 | 0.960 |  |
| rs891511 | A | G | 0.319 | -0.018 | 0.003 | 0.000 |  | 0.000 | 0.000 | 0.960 |  |
| rs11874246 | T | C | 0.294 | 0.017 | 0.003 | 0.000 |  | 0.000 | 0.000 | 0.970 |  |
| rs1250259 | A | T | 0.736 | -0.019 | 0.003 | 0.000 |  | 0.000 | 0.000 | 0.970 |  |

SBP, Systolic blood pressure; AF, Atrial fibrillation; SNP, single-nucleotide polymorphism; OR, odds ratio; CI, confidence interval.

*Exclude if P value <0.05.
