# Supplementary material for: Hypertension and Atrial Fibrillation: A Study on Epidemiology and Mendelian Randomization Causality
Source: Front Cardiovasc Med. 2021 Mar 23;8:644405. doi: 10.3389/fcvm.2021.644405 (PMC8021766; doi:10.3389/fcvm.2021.644405)
Supplement: Supplementary Table 3 — Characteristics of the SNPs associated with DBP and AF. [file Table_3.docx]

**Table Supplement 3 Characteristics of the SNPs associated with DBP and AF**

| SNP | EA | Other | EAF | Associations with DBP | | |  | Associations with AF | | | Management |
| --- | --- | --- | --- | --- | --- | --- | --- | --- | --- | --- | --- |
|  |  | allele |  | Beta | se | P value |  | Beta | se | P value |  |
| rs7173751 | A | G | 0.448 | 0.016 | 0.002 | 0.000 |  | 0.001 | 0.000 | 0.002 | Exclude |
| rs1363976 | C | T | 0.558 | -0.014 | 0.002 | 0.000 |  | -0.001 | 0.000 | 0.003 |  |
| rs10260816 | G | C | 0.438 | 0.018 | 0.002 | 0.000 |  | -0.001 | 0.000 | 0.004 |  |
| rs13125101 | A | G | 0.294 | 0.033 | 0.003 | 0.000 |  | 0.001 | 0.000 | 0.005 |  |
| rs7217916 | G | A | 0.615 | -0.015 | 0.003 | 0.000 |  | -0.001 | 0.000 | 0.006 |  |
| rs28545584 | A | G | 0.257 | -0.015 | 0.003 | 0.000 |  | -0.001 | 0.000 | 0.012 |  |
| rs58407878 | A | T | 0.140 | 0.020 | 0.004 | 0.000 |  | 0.001 | 0.000 | 0.012 |  |
| rs6026731 | T | C | 0.108 | 0.034 | 0.004 | 0.000 |  | 0.001 | 0.000 | 0.016 |  |
| rs916880 | A | G | 0.926 | 0.029 | 0.005 | 0.000 |  | 0.001 | 0.000 | 0.020 |  |
| rs12185567 | C | A | 0.719 | 0.016 | 0.003 | 0.000 |  | 0.001 | 0.000 | 0.021 |  |
| rs11021233 | A | G | 0.158 | -0.022 | 0.003 | 0.000 |  | -0.001 | 0.000 | 0.024 |  |
| rs11153730 | C | T | 0.493 | -0.014 | 0.002 | 0.000 |  | -0.001 | 0.000 | 0.024 |  |
| rs6490019 | G | A | 0.623 | 0.016 | 0.003 | 0.000 |  | 0.001 | 0.000 | 0.024 |  |
| rs6565192 | C | T | 0.467 | 0.016 | 0.002 | 0.000 |  | 0.001 | 0.000 | 0.028 |  |
| rs488834 | T | C | 0.767 | -0.017 | 0.003 | 0.000 |  | -0.001 | 0.000 | 0.032 |  |
| rs7837979 | T | C | 0.426 | 0.022 | 0.002 | 0.000 |  | 0.000 | 0.000 | 0.032 |  |
| rs11782222 | A | G | 0.221 | -0.026 | 0.003 | 0.000 |  | -0.001 | 0.000 | 0.034 |  |
| rs4835948 | C | T | 0.469 | 0.014 | 0.002 | 0.000 |  | 0.000 | 0.000 | 0.034 |  |
| rs28667801 | T | A | 0.406 | 0.015 | 0.003 | 0.000 |  | 0.000 | 0.000 | 0.035 |  |
| rs6585256 | C | A | 0.732 | 0.018 | 0.003 | 0.000 |  | 0.001 | 0.000 | 0.039 |  |
| rs13226502 | T | C | 0.177 | 0.023 | 0.003 | 0.000 |  | -0.001 | 0.000 | 0.047 |  |
| rs4305701 | A | C | 0.385 | 0.015 | 0.003 | 0.000 |  | 0.000 | 0.000 | 0.051 | include |
| rs351365 | C | T | 0.749 | 0.021 | 0.003 | 0.000 |  | 0.001 | 0.000 | 0.052 |  |
| rs116734066 | T | C | 0.093 | -0.024 | 0.004 | 0.000 |  | -0.001 | 0.000 | 0.053 |  |
| rs72677850 | A | G | 0.019 | -0.057 | 0.009 | 0.000 |  | 0.002 | 0.001 | 0.057 |  |
| rs34165865 | C | T | 0.198 | -0.018 | 0.003 | 0.000 |  | -0.001 | 0.000 | 0.058 |  |
| rs10840457 | G | A | 0.685 | -0.020 | 0.003 | 0.000 |  | 0.000 | 0.000 | 0.059 |  |
| rs7338606 | T | C | 0.216 | 0.020 | 0.003 | 0.000 |  | 0.001 | 0.000 | 0.067 |  |
| rs72812818 | C | G | 0.302 | -0.023 | 0.003 | 0.000 |  | 0.000 | 0.000 | 0.077 |  |
| rs12258967 | G | C | 0.298 | -0.026 | 0.003 | 0.000 |  | 0.000 | 0.000 | 0.087 |  |
| rs7500448 | G | A | 0.254 | 0.020 | 0.003 | 0.000 |  | 0.000 | 0.000 | 0.091 |  |
| rs604723 | C | T | 0.724 | 0.034 | 0.003 | 0.000 |  | 0.000 | 0.000 | 0.093 |  |
| rs61148001 | T | C | 0.211 | -0.018 | 0.003 | 0.000 |  | 0.000 | 0.000 | 0.097 |  |
| rs60991988 | G | T | 0.104 | 0.025 | 0.004 | 0.000 |  | -0.001 | 0.000 | 0.100 |  |
| rs62107917 | A | G | 0.168 | 0.019 | 0.003 | 0.000 |  | 0.001 | 0.000 | 0.100 |  |
| rs34483452 | A | C | 0.135 | 0.022 | 0.004 | 0.000 |  | 0.001 | 0.000 | 0.110 |  |
| rs7199293 | A | G | 0.529 | 0.014 | 0.002 | 0.000 |  | 0.000 | 0.000 | 0.110 |  |
| rs55857306 | A | G | 0.164 | -0.039 | 0.003 | 0.000 |  | 0.000 | 0.000 | 0.120 |  |
| rs2207231 | G | A | 0.105 | -0.023 | 0.004 | 0.000 |  | 0.001 | 0.000 | 0.130 |  |
| rs35444 | G | A | 0.383 | -0.020 | 0.003 | 0.000 |  | 0.000 | 0.000 | 0.130 |  |
| rs4507656 | G | C | 0.305 | 0.015 | 0.003 | 0.000 |  | 0.000 | 0.000 | 0.130 |  |
| rs1436138 | G | A | 0.358 | -0.016 | 0.003 | 0.000 |  | 0.000 | 0.000 | 0.140 |  |
| rs6804765 | G | A | 0.583 | 0.017 | 0.002 | 0.000 |  | 0.000 | 0.000 | 0.140 |  |
| rs7613528 | T | C | 0.263 | 0.017 | 0.003 | 0.000 |  | 0.000 | 0.000 | 0.140 |  |
| rs2524080 | T | A | 0.676 | 0.023 | 0.003 | 0.000 |  | 0.000 | 0.000 | 0.150 |  |
| rs836179 | G | A | 0.368 | 0.020 | 0.003 | 0.000 |  | 0.000 | 0.000 | 0.150 |  |
| rs56206297 | T | G | 0.163 | 0.021 | 0.003 | 0.000 |  | 0.000 | 0.000 | 0.160 |  |
| rs13293465 | T | C | 0.352 | -0.015 | 0.003 | 0.000 |  | 0.000 | 0.000 | 0.170 |  |
| rs34004783 | G | C | 0.658 | -0.021 | 0.003 | 0.000 |  | 0.000 | 0.000 | 0.170 |  |
| rs115262049 | T | A | 0.089 | -0.025 | 0.004 | 0.000 |  | -0.001 | 0.000 | 0.180 |  |
| rs72854462 | G | A | 0.251 | 0.025 | 0.003 | 0.000 |  | 0.000 | 0.000 | 0.190 |  |
| rs77924615 | A | G | 0.198 | -0.023 | 0.003 | 0.000 |  | 0.000 | 0.000 | 0.190 |  |
| rs59549190 | A | G | 0.340 | -0.018 | 0.003 | 0.000 |  | 0.000 | 0.000 | 0.200 |  |
| rs7911644 | T | C | 0.329 | 0.016 | 0.003 | 0.000 |  | 0.000 | 0.000 | 0.200 |  |
| rs6271 | T | C | 0.074 | -0.036 | 0.005 | 0.000 |  | 0.001 | 0.000 | 0.210 |  |
| rs74444983 | C | T | 0.260 | 0.017 | 0.003 | 0.000 |  | 0.000 | 0.000 | 0.220 |  |
| rs11677058 | G | A | 0.463 | -0.014 | 0.002 | 0.000 |  | 0.000 | 0.000 | 0.230 |  |
| rs78845099 | T | A | 0.276 | -0.028 | 0.003 | 0.000 |  | 0.000 | 0.000 | 0.230 |  |
| rs9577891 | T | C | 0.470 | -0.013 | 0.002 | 0.000 |  | 0.000 | 0.000 | 0.230 |  |
| rs13107325 | T | C | 0.075 | -0.042 | 0.005 | 0.000 |  | 0.001 | 0.000 | 0.240 |  |
| rs1353776 | G | C | 0.645 | -0.014 | 0.003 | 0.000 |  | 0.000 | 0.000 | 0.240 |  |
| rs167479 | T | G | 0.470 | -0.027 | 0.002 | 0.000 |  | 0.000 | 0.000 | 0.250 |  |
| rs35552228 | G | T | 0.246 | 0.017 | 0.003 | 0.000 |  | 0.000 | 0.000 | 0.250 |  |
| rs6460541 | G | A | 0.262 | 0.018 | 0.003 | 0.000 |  | 0.000 | 0.000 | 0.250 |  |
| rs11191580 | C | T | 0.077 | -0.038 | 0.005 | 0.000 |  | 0.000 | 0.000 | 0.260 |  |
| rs72834627 | C | G | 0.121 | 0.028 | 0.004 | 0.000 |  | 0.000 | 0.000 | 0.260 |  |
| rs889509 | G | A | 0.417 | -0.021 | 0.003 | 0.000 |  | 0.000 | 0.000 | 0.260 |  |
| rs2301597 | C | T | 0.576 | -0.017 | 0.002 | 0.000 |  | 0.000 | 0.000 | 0.270 |  |
| rs2782980 | C | T | 0.722 | 0.026 | 0.003 | 0.000 |  | 0.000 | 0.000 | 0.270 |  |
| rs28406364 | T | C | 0.377 | 0.017 | 0.003 | 0.000 |  | 0.000 | 0.000 | 0.270 |  |
| rs73030276 | T | C | 0.270 | -0.016 | 0.003 | 0.000 |  | 0.000 | 0.000 | 0.270 |  |
| rs10195405 | T | A | 0.290 | -0.016 | 0.003 | 0.000 |  | 0.000 | 0.000 | 0.280 |  |
| rs111346856 | A | G | 0.277 | 0.017 | 0.003 | 0.000 |  | 0.000 | 0.000 | 0.290 |  |
| rs39281 | G | A | 0.532 | -0.016 | 0.002 | 0.000 |  | 0.000 | 0.000 | 0.300 |  |
| rs4236489 | A | G | 0.505 | 0.014 | 0.002 | 0.000 |  | 0.000 | 0.000 | 0.300 |  |
| rs66887589 | C | T | 0.473 | 0.015 | 0.002 | 0.000 |  | 0.000 | 0.000 | 0.300 |  |
| rs12656497 | C | T | 0.596 | 0.025 | 0.003 | 0.000 |  | 0.000 | 0.000 | 0.310 |  |
| rs12905116 | A | T | 0.760 | 0.019 | 0.003 | 0.000 |  | 0.000 | 0.000 | 0.310 |  |
| rs75531890 | T | C | 0.060 | 0.033 | 0.005 | 0.000 |  | 0.000 | 0.000 | 0.310 |  |
| rs2493296 | T | C | 0.137 | 0.028 | 0.004 | 0.000 |  | 0.000 | 0.000 | 0.320 |  |
| rs7373878 | T | G | 0.621 | -0.014 | 0.003 | 0.000 |  | 0.000 | 0.000 | 0.320 |  |
| rs2979666 | C | A | 0.514 | 0.015 | 0.002 | 0.000 |  | 0.000 | 0.000 | 0.330 |  |
| rs62217514 | T | C | 0.745 | 0.019 | 0.003 | 0.000 |  | 0.000 | 0.000 | 0.330 |  |
| rs7129204 | C | G | 0.120 | 0.026 | 0.004 | 0.000 |  | 0.000 | 0.000 | 0.330 |  |
| rs1078721 | T | C | 0.202 | -0.020 | 0.003 | 0.000 |  | 0.000 | 0.000 | 0.340 |  |
| rs12454132 | G | A | 0.328 | -0.017 | 0.003 | 0.000 |  | 0.000 | 0.000 | 0.340 |  |
| rs73033340 | G | A | 0.032 | -0.041 | 0.007 | 0.000 |  | 0.001 | 0.001 | 0.340 |  |
| rs78473917 | C | T | 0.149 | -0.023 | 0.003 | 0.000 |  | 0.000 | 0.000 | 0.340 |  |
| rs3789592 | G | A | 0.560 | 0.017 | 0.002 | 0.000 |  | 0.000 | 0.000 | 0.350 |  |
| rs1035673 | C | T | 0.601 | -0.015 | 0.003 | 0.000 |  | 0.000 | 0.000 | 0.360 |  |
| rs72936986 | C | A | 0.279 | -0.019 | 0.003 | 0.000 |  | 0.000 | 0.000 | 0.360 |  |
| rs208078 | T | G | 0.633 | 0.015 | 0.003 | 0.000 |  | 0.000 | 0.000 | 0.370 |  |
| rs2274224 | C | G | 0.432 | -0.023 | 0.002 | 0.000 |  | 0.000 | 0.000 | 0.390 |  |
| rs11718573 | C | G | 0.501 | 0.015 | 0.002 | 0.000 |  | 0.000 | 0.000 | 0.400 |  |
| rs2306363 | T | G | 0.207 | -0.022 | 0.003 | 0.000 |  | 0.000 | 0.000 | 0.400 |  |
| rs2836411 | T | C | 0.354 | 0.020 | 0.003 | 0.000 |  | 0.000 | 0.000 | 0.400 |  |
| rs4923915 | T | C | 0.336 | 0.019 | 0.003 | 0.000 |  | 0.000 | 0.000 | 0.400 |  |
| rs6708660 | C | T | 0.404 | -0.017 | 0.003 | 0.000 |  | 0.000 | 0.000 | 0.400 |  |
| rs9296668 | G | A | 0.484 | 0.018 | 0.002 | 0.000 |  | 0.000 | 0.000 | 0.400 |  |
| rs9548033 | C | T | 0.430 | 0.014 | 0.003 | 0.000 |  | 0.000 | 0.000 | 0.400 |  |
| rs149453951 | T | C | 0.024 | -0.073 | 0.008 | 0.000 |  | -0.001 | 0.001 | 0.410 |  |
| rs113044050 | T | C | 0.141 | -0.020 | 0.004 | 0.000 |  | 0.000 | 0.000 | 0.420 |  |
| rs6487076 | G | A | 0.224 | -0.016 | 0.003 | 0.000 |  | 0.000 | 0.000 | 0.430 |  |
| rs1980235 | A | G | 0.673 | 0.021 | 0.003 | 0.000 |  | 0.000 | 0.000 | 0.440 |  |
| rs145681012 | T | G | 0.020 | 0.053 | 0.009 | 0.000 |  | -0.001 | 0.001 | 0.480 |  |
| rs9352694 | G | A | 0.390 | 0.018 | 0.003 | 0.000 |  | 0.000 | 0.000 | 0.480 |  |
| rs6001795 | A | G | 0.203 | -0.017 | 0.003 | 0.000 |  | 0.000 | 0.000 | 0.490 |  |
| rs7258382 | C | T | 0.164 | -0.019 | 0.003 | 0.000 |  | 0.000 | 0.000 | 0.490 |  |
| rs12693302 | A | G | 0.649 | -0.023 | 0.003 | 0.000 |  | 0.000 | 0.000 | 0.500 |  |
| rs1446468 | C | T | 0.546 | 0.018 | 0.002 | 0.000 |  | 0.000 | 0.000 | 0.500 |  |
| rs35213536 | T | G | 0.243 | 0.019 | 0.003 | 0.000 |  | 0.000 | 0.000 | 0.500 |  |
| rs72654647 | A | G | 0.245 | 0.017 | 0.003 | 0.000 |  | 0.000 | 0.000 | 0.500 |  |
| rs741066 | T | C | 0.299 | 0.016 | 0.003 | 0.000 |  | 0.000 | 0.000 | 0.500 |  |
| rs76438269 | G | A | 0.062 | 0.032 | 0.005 | 0.000 |  | 0.000 | 0.000 | 0.510 |  |
| rs597808 | G | A | 0.517 | -0.038 | 0.002 | 0.000 |  | 0.000 | 0.000 | 0.520 |  |
| rs8081273 | T | C | 0.376 | -0.015 | 0.003 | 0.000 |  | 0.000 | 0.000 | 0.520 |  |
| rs12712691 | T | C | 0.468 | -0.014 | 0.002 | 0.000 |  | 0.000 | 0.000 | 0.530 |  |
| rs12906962 | C | T | 0.318 | 0.022 | 0.003 | 0.000 |  | 0.000 | 0.000 | 0.530 |  |
| rs9689079 | G | A | 0.780 | -0.017 | 0.003 | 0.000 |  | 0.000 | 0.000 | 0.530 |  |
| rs71532343 | C | T | 0.172 | 0.020 | 0.003 | 0.000 |  | 0.000 | 0.000 | 0.540 |  |
| rs1530558 | C | T | 0.136 | -0.021 | 0.004 | 0.000 |  | 0.000 | 0.000 | 0.550 |  |
| rs2014590 | T | C | 0.487 | -0.024 | 0.002 | 0.000 |  | 0.000 | 0.000 | 0.550 |  |
| rs3812 | C | G | 0.627 | -0.015 | 0.003 | 0.000 |  | 0.000 | 0.000 | 0.560 |  |
| rs9773022 | C | T | 0.460 | 0.014 | 0.002 | 0.000 |  | 0.000 | 0.000 | 0.560 |  |
| rs73606412 | T | C | 0.157 | -0.024 | 0.003 | 0.000 |  | 0.000 | 0.000 | 0.570 |  |
| rs10975462 | G | A | 0.231 | 0.016 | 0.003 | 0.000 |  | 0.000 | 0.000 | 0.590 |  |
| rs11145807 | G | A | 0.595 | -0.016 | 0.003 | 0.000 |  | 0.000 | 0.000 | 0.590 |  |
| rs3851374 | G | A | 0.820 | 0.018 | 0.003 | 0.000 |  | 0.000 | 0.000 | 0.590 |  |
| rs1872167 | T | C | 0.140 | -0.022 | 0.004 | 0.000 |  | 0.000 | 0.000 | 0.600 |  |
| rs66682451 | G | A | 0.277 | -0.017 | 0.003 | 0.000 |  | 0.000 | 0.000 | 0.600 |  |
| rs11556924 | T | C | 0.390 | -0.016 | 0.003 | 0.000 |  | 0.000 | 0.000 | 0.610 |  |
| rs6089970 | T | C | 0.232 | -0.016 | 0.003 | 0.000 |  | 0.000 | 0.000 | 0.610 |  |
| rs35807464 | A | G | 0.066 | 0.036 | 0.005 | 0.000 |  | 0.000 | 0.000 | 0.620 |  |
| rs2049114 | C | T | 0.794 | 0.022 | 0.003 | 0.000 |  | 0.000 | 0.000 | 0.630 |  |
| rs507666 | A | G | 0.186 | -0.021 | 0.003 | 0.000 |  | 0.000 | 0.000 | 0.630 |  |
| rs11222084 | T | A | 0.372 | -0.023 | 0.003 | 0.000 |  | 0.000 | 0.000 | 0.640 |  |
| rs12494396 | C | G | 0.667 | 0.017 | 0.003 | 0.000 |  | 0.000 | 0.000 | 0.660 |  |
| rs28661492 | T | C | 0.192 | -0.018 | 0.003 | 0.000 |  | 0.000 | 0.000 | 0.660 |  |
| rs56256623 | A | C | 0.187 | -0.021 | 0.003 | 0.000 |  | 0.000 | 0.000 | 0.660 |  |
| rs12324159 | A | G | 0.430 | 0.017 | 0.002 | 0.000 |  | 0.000 | 0.000 | 0.670 |  |
| rs810057 | C | T | 0.338 | 0.019 | 0.003 | 0.000 |  | 0.000 | 0.000 | 0.670 |  |
| rs2238435 | G | C | 0.616 | 0.014 | 0.003 | 0.000 |  | 0.000 | 0.000 | 0.680 |  |
| rs997988 | A | T | 0.400 | 0.014 | 0.003 | 0.000 |  | 0.000 | 0.000 | 0.690 |  |
| rs4476839 | A | G | 0.871 | -0.021 | 0.004 | 0.000 |  | 0.000 | 0.000 | 0.700 |  |
| rs4919493 | C | T | 0.887 | 0.023 | 0.004 | 0.000 |  | 0.000 | 0.000 | 0.700 |  |
| rs35479618 | A | G | 0.017 | 0.067 | 0.009 | 0.000 |  | 0.000 | 0.001 | 0.730 |  |
| rs504691 | A | C | 0.397 | -0.015 | 0.003 | 0.000 |  | 0.000 | 0.000 | 0.730 |  |
| rs9834975 | T | A | 0.444 | 0.015 | 0.002 | 0.000 |  | 0.000 | 0.000 | 0.730 |  |
| rs1450271 | T | C | 0.472 | 0.020 | 0.002 | 0.000 |  | 0.000 | 0.000 | 0.740 |  |
| rs1509966 | A | G | 0.499 | -0.021 | 0.002 | 0.000 |  | 0.000 | 0.000 | 0.740 |  |
| rs2305758 | T | C | 0.283 | -0.015 | 0.003 | 0.000 |  | 0.000 | 0.000 | 0.740 |  |
| rs4076877 | T | C | 0.054 | -0.033 | 0.006 | 0.000 |  | 0.000 | 0.001 | 0.740 |  |
| rs882624 | T | C | 0.333 | -0.019 | 0.003 | 0.000 |  | 0.000 | 0.000 | 0.740 |  |
| rs3827750 | T | C | 0.111 | 0.028 | 0.004 | 0.000 |  | 0.000 | 0.000 | 0.760 |  |
| rs7799613 | G | C | 0.599 | -0.014 | 0.003 | 0.000 |  | 0.000 | 0.000 | 0.790 |  |
| rs6771928 | A | G | 0.144 | 0.033 | 0.004 | 0.000 |  | 0.000 | 0.000 | 0.800 |  |
| rs1579036 | A | G | 0.319 | 0.019 | 0.003 | 0.000 |  | 0.000 | 0.000 | 0.810 |  |
| rs11636952 | C | T | 0.695 | -0.029 | 0.003 | 0.000 |  | 0.000 | 0.000 | 0.820 |  |
| rs60687229 | C | T | 0.140 | 0.020 | 0.004 | 0.000 |  | 0.000 | 0.000 | 0.820 |  |
| rs6983488 | A | C | 0.361 | -0.014 | 0.003 | 0.000 |  | 0.000 | 0.000 | 0.820 |  |
| rs134041 | C | T | 0.562 | -0.015 | 0.002 | 0.000 |  | 0.000 | 0.000 | 0.830 |  |
| rs4762921 | A | G | 0.718 | 0.026 | 0.003 | 0.000 |  | 0.000 | 0.000 | 0.830 |  |
| rs6923947 | A | G | 0.447 | 0.022 | 0.002 | 0.000 |  | 0.000 | 0.000 | 0.830 |  |
| rs7200432 | A | G | 0.300 | -0.015 | 0.003 | 0.000 |  | 0.000 | 0.000 | 0.850 |  |
| rs6770911 | C | A | 0.705 | 0.017 | 0.003 | 0.000 |  | 0.000 | 0.000 | 0.870 |  |
| rs35346340 | C | G | 0.327 | 0.027 | 0.003 | 0.000 |  | 0.000 | 0.000 | 0.880 |  |
| rs72831343 | G | T | 0.146 | -0.033 | 0.003 | 0.000 |  | 0.000 | 0.000 | 0.900 |  |
| rs343011 | G | T | 0.766 | -0.021 | 0.003 | 0.000 |  | 0.000 | 0.000 | 0.910 |  |
| rs3943093 | T | C | 0.322 | 0.026 | 0.003 | 0.000 |  | 0.000 | 0.000 | 0.910 |  |
| rs2782652 | C | T | 0.454 | 0.016 | 0.002 | 0.000 |  | 0.000 | 0.000 | 0.920 |  |
| rs650724 | A | G | 0.092 | -0.025 | 0.004 | 0.000 |  | 0.000 | 0.000 | 0.930 |  |
| rs673791 | G | C | 0.621 | 0.017 | 0.003 | 0.000 |  | 0.000 | 0.000 | 0.930 |  |
| rs8111686 | A | G | 0.330 | 0.023 | 0.003 | 0.000 |  | 0.000 | 0.000 | 0.930 |  |
| rs7744284 | T | C | 0.522 | -0.014 | 0.002 | 0.000 |  | 0.000 | 0.000 | 0.950 |  |
| rs78378222 | G | T | 0.013 | 0.068 | 0.011 | 0.000 |  | 0.000 | 0.001 | 0.950 |  |
| rs3118904 | A | G | 0.352 | 0.014 | 0.003 | 0.000 |  | 0.000 | 0.000 | 0.960 |  |
| rs891511 | A | G | 0.319 | -0.024 | 0.003 | 0.000 |  | 0.000 | 0.000 | 0.960 |  |
| rs17717829 | C | T | 0.455 | -0.016 | 0.002 | 0.000 |  | 0.000 | 0.000 | 0.990 |  |
| rs34039016 | A | G | 0.267 | 0.018 | 0.003 | 0.000 |  | 0.000 | 0.000 | 0.990 |  |
| rs185373991 | A | T | 0.121 | 0.021 | 0.004 | 0.000 |  | 0.000 | 0.000 | 1.000 |  |
| rs751984 | C | T | 0.115 | -0.035 | 0.004 | 0.000 |  | 0.000 | 0.000 | 1.000 |  |

DBP, Diastolic blood pressure; AF, Atrial fibrillation; SNP, single-nucleotide polymorphism; OR, odds ratio; CI, confidence interval.
