# Supplementary material for: Hypertension and Atrial Fibrillation: A Study on Epidemiology and Mendelian Randomization Causality
Source: Front Cardiovasc Med. 2021 Mar 23;8:644405. doi: 10.3389/fcvm.2021.644405 (PMC8021766; doi:10.3389/fcvm.2021.644405)
Supplement: Supplementary Table 4 — Baseline characteristic of study populations by hypertension. [file Table_4.docx]

**Table** **Supplement 4 Baseline characteristic of study populations by hypertension**

| Characteristic | Total  (n=9474) | Hypertension  (n=1190) | Non-hypertension  (n=8284) | P value |
| --- | --- | --- | --- | --- |
| Age, years | 53.7±5.7 | 55.5±5.7 | 53.4±5.7 | <0.001 |
| Male | 4494 (47.4%) | 641 (53.9%) | 3853 (46.5%) | <0.001 |
| White | 7644 (80.7%) | 721 (60.6%) | 6023 (83.6%) | <0.001 |
| BMI, kg/m^2^ | 27.8±4.8 | 28.4±5.7 | 26.6±4.6 | <0.001 |
| SBP, mmHg | 118.2±17.5 | 149.9±15.4 | 113.7±12.3 | <0.001 |
| DBP, mmHg | 72.2±10.7 | 87.0±11.6 | 70.0±8.7 | <0.001 |
| Sport | 2.5±0.8 | 2.4±0.8 | 2.5±0.8 | <0.001 |
| Smoking |  |  |  | 0.444 |
| Never | 3826 (40.4%) | 500 (42.0%) | 3326 (40.1%) |  |
| Former | 3084 (32.6%) | 381 (32.0%) | 2703 (32.6%) |  |
| Current | 2564 (27.1%) | 309 (26.0%) | 2255 (27.2%) |  |
| Drinking |  |  |  | <0.001 |
| Never | 2042 (21.6%) | 311 (26.1%) | 1731 (20.9%) |  |
| Former | 1630 (17.2%) | 214 (18.0%) | 1416 (17.1%) |  |
| Current | 5802 (61.2%) | 665 (55.9%) | 5137 (62.0%) |  |
| Education |  |  |  | <0.001 |
| Basic | 1849 (19.4%) | 353 (29.7%) | 1487 (18.0%) |  |
| Intermediate | 3994 (42.2%) | 463 (38.9%) | 3531 (42.6%) |  |
| Advanced | 3640 (38.4%) | 374 (31.4%) | 3266 (39.4%) |  |
| Creatine, mg/dl | 1.1±0.4 | 1.1±0.7 | 1.1±0.3 | <0.001 |
| HDL-c, mmol/L | 1.4±0.4 | 1.4±0.5 | 1.4±0.4 | 0.869 |
| LDL-c, mmol/L | 3.5±1.0 | 3.6±1.0 | 3.5±1.0 | 0.001 |
| TC, mmol/L | 5.5±1.0 | 5.6±1.1 | 5.5±1.0 | <0.001 |
| TG, mmol/L | 1.3±0.7 | 1.4±0.8 | 1.3±0.7 | <0.001 |
| Glucose, mmol/L | 5.8±1.7 | 6.2±2.6 | 5.7±1.6 | <0.001 |
| HF | 15 (0.2%) | 2 (0.2%) | 13 (0.2%) | 1.00 |
| CHD | 205 (2.2%) | 25 (2.1%) | 180 (2.2%) | 0.873 |
| Diabetes | 548 (5.8%) | 122 (10.3%) | 426 (5.1%) | <0.001 |
| Stain | 30 (0.3%) | 4 (0.3%) | 26 (0.3%) | 0.785 |
| Aspirin | 4273 (45.1%) | 486 (40.8%) | 3787 (45.7%) | 0.002 |
| Anticoagulants | 17 (0.2%) | 4 (0.3%) | 13 (0.2%) | 0.258 |

BMI = body mass index; SBP = systolic blood pressure; DBP = diastolic blood pressure; HDL-c = high-density lipoprotein cholesterol; LDL-c = low-density lipoprotein cholesterol; TC = total cholesterol; TG = triglyceride; HF = heart failure; CHD = coronary heart disease.
