# Supplementary material for: Hypertension and Atrial Fibrillation: A Study on Epidemiology and Mendelian Randomization Causality
Source: Front Cardiovasc Med. 2021 Mar 23;8:644405. doi: 10.3389/fcvm.2021.644405 (PMC8021766; doi:10.3389/fcvm.2021.644405)
Supplement: Supplementary Table 5 — Hazard ratio of HT for AF. [file Table_5.docx]

**Table Supplement 5 Hazard ratio of HT for AF**

| Risk factor | Event rates | Model 1 | |  | Model 2 | |  | Model 3 | |
| --- | --- | --- | --- | --- | --- | --- | --- | --- | --- |
|  |  | HR (95% CI) | P value |  | HR (95% CI) | P value |  | HR (95% CI) | P value |
| HT | 1414/9474 | 1.57 (1.36, 1.81) | <0.001 |  | 1.50 (1.30, 1.73) | <0.001 |  | 1.50 (1.29, 1.73) | <0.001 |
| SBP, per 10 mmHg | 1414/9474 | 1.14 (1.11, 1.17) | <0.001 |  | 1.12 (1.09, 1.15) | <0.001 |  | 1.17 (1.12, 1.22)^*^ | <0.001 |
| DBP, per 10 mmHg | 1414/9474 | 1.07 (1.02, 1.13) | 0.009 |  | 1.08 (1.01, 1.13) | <0.015 |  | 0.90 (0.84, 0.97)^#^ | 0.005 |

Model 1: adjusted for gender, race, age;

Model 2: further adjusted for BMI, smoking, drinking, education level, sport, heart failure, coronary heart disease, diabetes;

Model 3: further adjusted for creatine, HDL-c, LDL-c, TG, glucose, stain, aspirin, anticoagulants.

^*^Further adjusted for DBP;^#^Further adjusted for SBP.

HR, Hazard ratio; HT, Hypertension; AF, Atrial fibrillation; BMI, Body mass index; SBP, Systolic blood pressure; DBP, Diastolic blood pressure; HDL-c, High-density lipoprotein cholesterol; LDL-c, Low-density lipoprotein cholesterol; TG, Triglyceride.
