# Supplementary material for: Hypertension and Atrial Fibrillation: A Study on Epidemiology and Mendelian Randomization Causality
Source: Front Cardiovasc Med. 2021 Mar 23;8:644405. doi: 10.3389/fcvm.2021.644405 (PMC8021766; doi:10.3389/fcvm.2021.644405)
Supplement: Supplementary Table 6 — Causal associations between genetically determined HT, SBP, DBP and AF. [file Table_6.docx]

**Table Supplement 6 Causal associations between genetically determined HT, SBP, DBP and AF**

| Exposure-outcome | Method |  | Causal estimate | | | | |
| --- | --- | --- | --- | --- | --- | --- | --- |
|  |  | SNP | | OR | 95%CI | | P value |
| HT→AF | MR Egger | 3 | | 1.92 | 0.82 | 4.51 | 0.38 |
|  | Weighted median | 3 | | 1.71 | 0.97 | 3.02 | 0.06 |
|  | IVW | 3 | | 1.90 | 1.18 | 3.04 | 0.01 |
|  | Weighted mode | 3 | | 1.67 | 0.87 | 3.22 | 0.27 |
| Test for Heterogeneity: P = 0.42 (MR-Egger) and P =0.72 (IVW) | | | | | | |  |
| Test for Horizontal pleiotropy: MR-Egger intercept =-0.000018, se=0.00055, P=0.97 | | | | | | |  |
| SBP→AF | MR Egger | 142 | | 1.05 | 0.98 | 1.12 | 0.13 |
|  | Weighted median | 142 | | 1.04 | 1.01 | 1.07 | 0.02 |
|  | IVW | 142 | | 1.03 | 1.01 | 1.05 | 0.01 |
|  | Weighted mode | 142 | | 1.08 | 1.00 | 1.17 | 0.05 |
| Test for Heterogeneity: P = 0.88 (MR-Egger) and P =0.88 (IVW) | | | | | | |  |
| Test for Horizontal pleiotropy: MR-Egger intercept =-0.000053, se=0.000072, P=0.46 | | | | | | |  |
| DBP→AF | MR Egger | 157 | | 1.00 | 0.93 | 1.07 | 1.00 |
|  | Weighted median | 157 | | 1.02 | 0.99 | 1.06 | 0.11 |
|  | IVW | 157 | | 1.02 | 1.00 | 1.04 | 0.03 |
|  | Weighted mode | 157 | | 1.09 | 1.02 | 1.17 | 0.02 |
| Test for Heterogeneity: P = 0.72 (MR-Egger) and P =0.73 (IVW) | | | | | | |  |
| Test for Horizontal pleiotropy: MR-Egger intercept =0.000049, se=0.000074, P=0.50 | | | | | | |  |

HT, Hypertension; SBP, Systolic blood pressure (10 mmHg); DBP, Diastolic blood pressure (10 mmHg); AF, Atrial fibrillation; SNP, single-nucleotide polymorphism; OR, Odds ratio; CI, Confidence interval; Inverse variance weighted, IVW.
